# Supplementary material for: Novel D-A-π-A1 Type Organic Sensitizers from 4,7-Dibromobenzo[d][1,2,3]thiadiazole and Indoline Donors for Dye-Sensitized Solar Cells
Source: Molecules. 2022 Jun 29;27(13):4197. doi: 10.3390/molecules27134197 (PMC9267993; doi:10.3390/molecules27134197)
Supplement: Supplementary file 1 [file molecules-27-04197-s001.zip › molecules-1767494-supplementary.pdf]

Supplementary Materials

Novel D-A-π-A1 type organic sensitizers from 4,7-dibromobenzo[*d*][1,2,3]thiadiazole and indoline donors for dye-sensitized solar cells

Nikita S. Gudim [1], Ekaterina A. Knyazeva [1,2], Ludmila V. Mihalchenko [1], Maksim S. Mikhailov [2], Lu Zhang [3], Neil Robertson,[3]* and Oleg A. Rakitin [1,*]

[1] N. D. Zelinsky Institute of Organic Chemistry, Russian Academy of Sciences, 119991 Moscow, Russian Federation; orakitin@ioc.ac.ru (O.A.R.); nikitosgudim@gmail.com (N.S.G.); katerina_knyazev@ioc.ac.ru (E.A.K.)
[2] Nanotechnology Education and Research Center, South Ural State University, 454080 Chelyabinsk, Russia; katerina_knyazev@mail.ru (E.A.K.)
[3] EaStCHEM School of Chemistry, University of Edinburgh, Edinburgh EH9 3FJ, UK; neil.robertson@ed.ac.uk (N.R.)
***** Correspondence: neil.robertson@ed.ac.uk; orakitin@ioc.ac.ru; Tel.: +7 499 135 5327

Table of Contents

1. **NMR spectra**
2. **Crystal data and structure refinement for 7e, 7f, 7b, and 8b**
3. **Statistics of photovoltaic performance of DSSCs fabricated with KEA dyes**
4. **General procedure for fabrication and characterization of DSSCs**

1. NMR spectra

7-Bromo-4-(9-hexyl-9*H*-carbazol-3-yl)benzo[*d*][1,2,3]thiadiazole (7a)

^{1}H NMR (300 MHz)

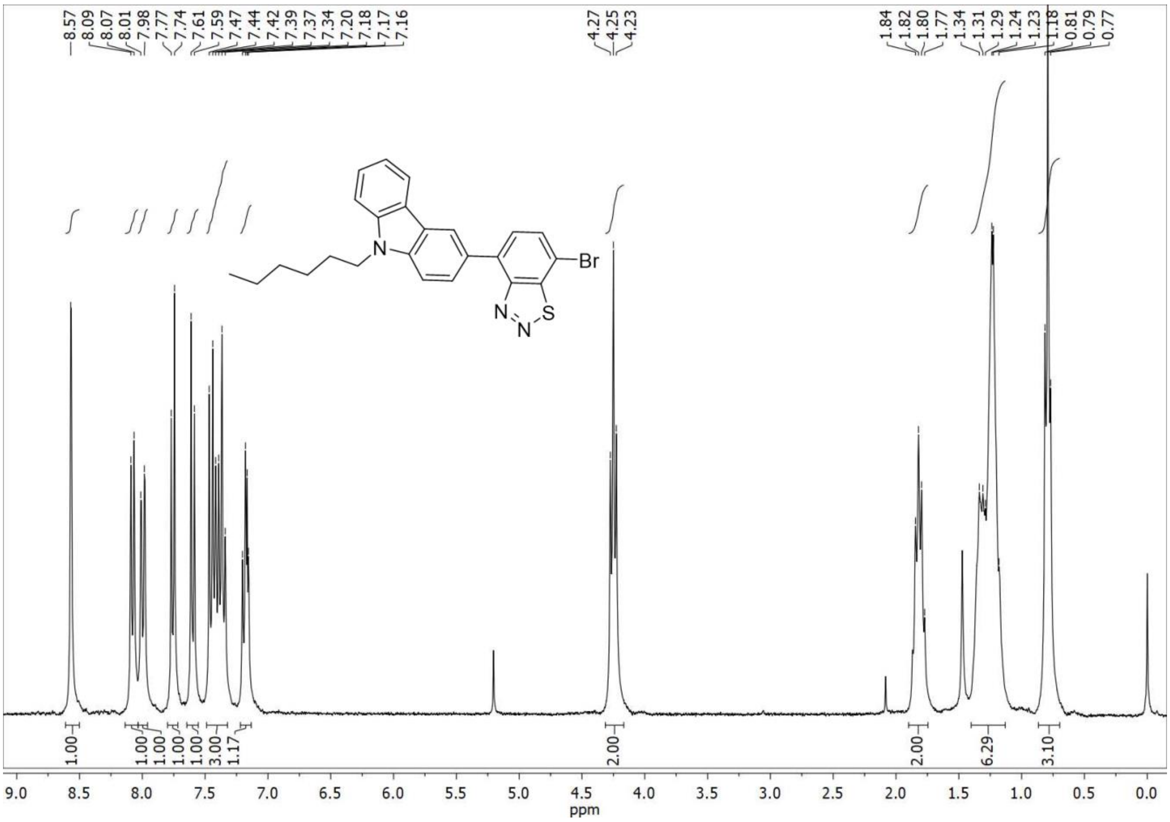

^{13}C NMR (75 MHz)

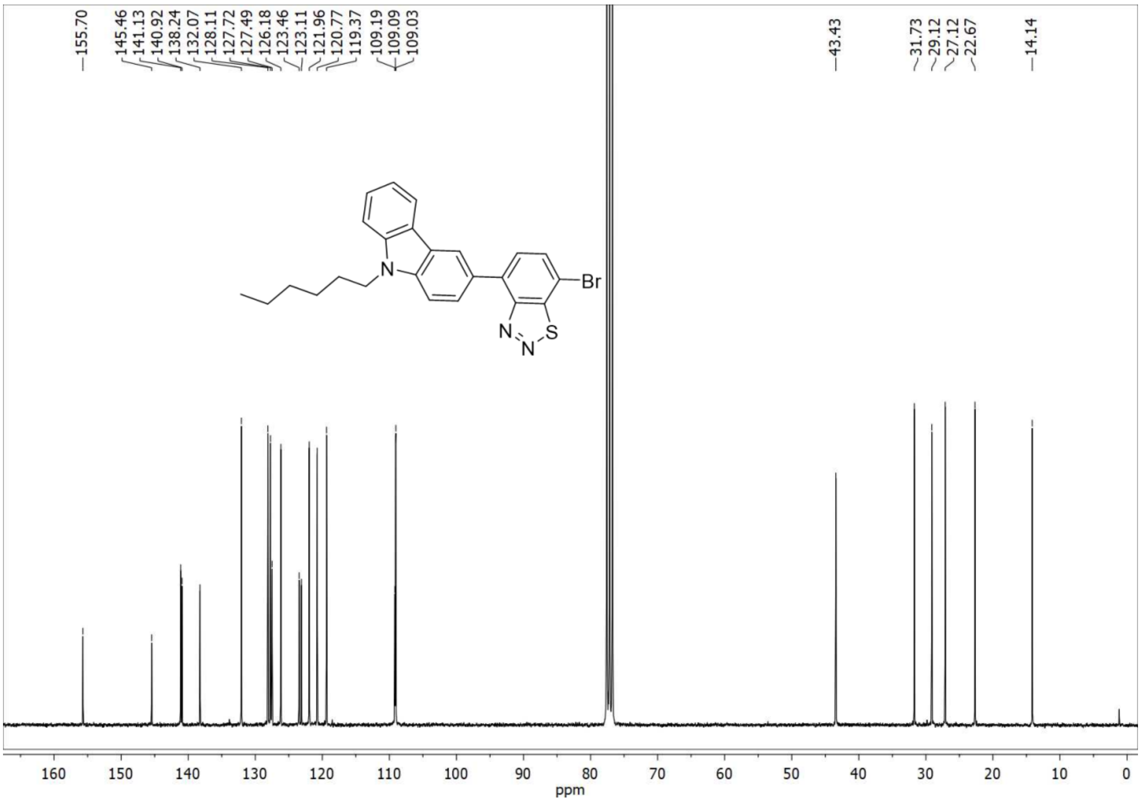

4-Bromo-7-(9-hexyl-9*H*-carbazol-3-yl)benzo[*d*][1,2,3]thiadiazole (8a)

^{1}H NMR (300 MHz)

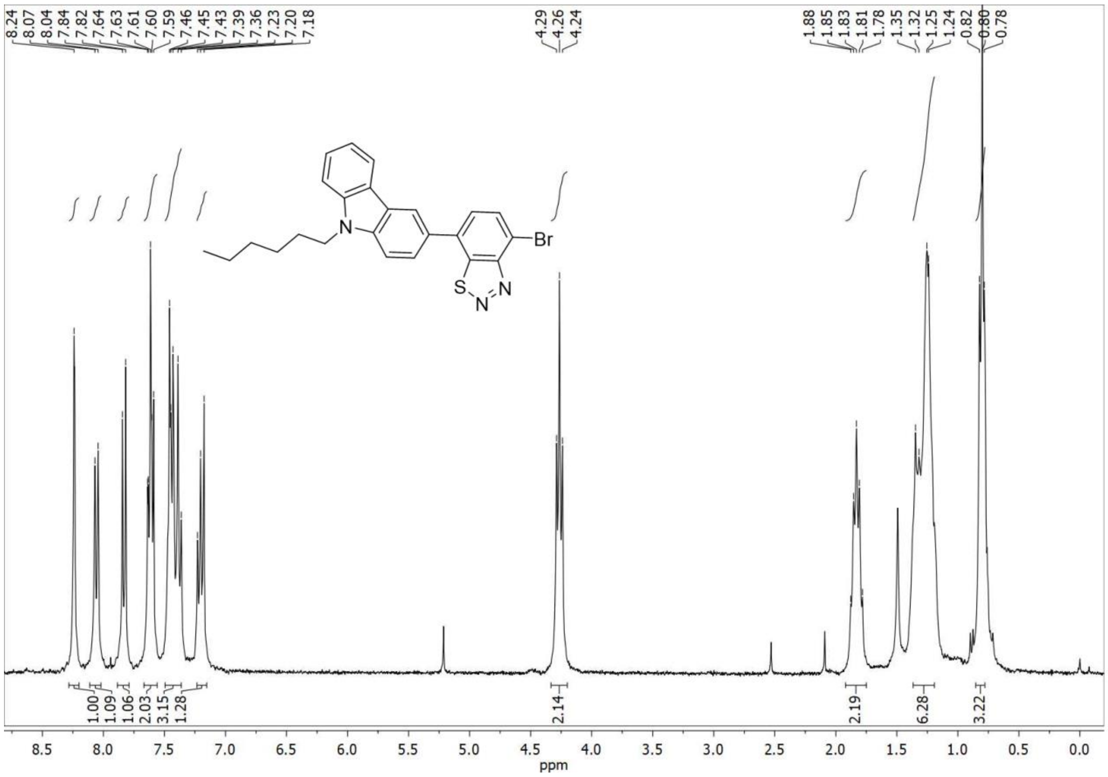

^{13}C NMR (75 MHz)

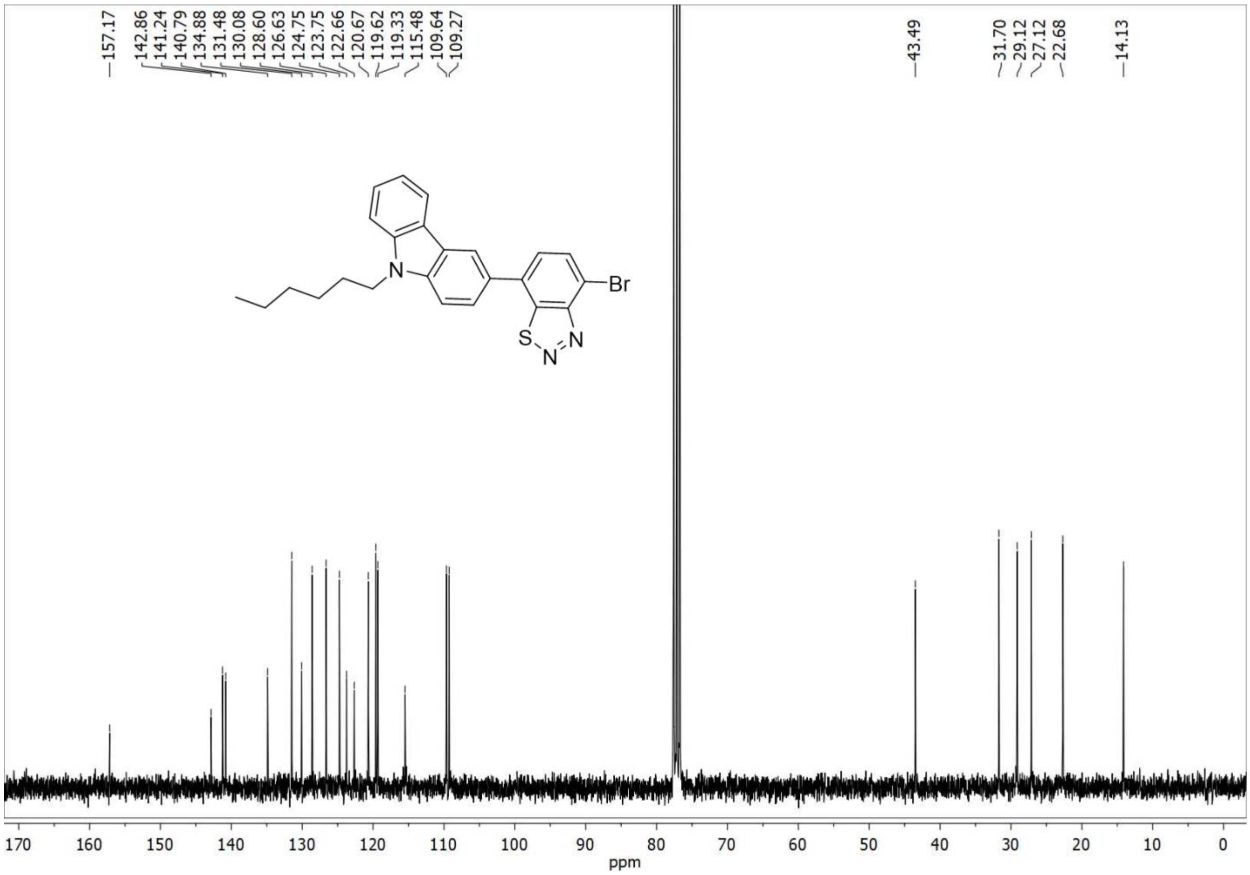

4,7-Bis(9-hexyl-9*H*-carbazol-3-yl)benzo[*d*][1,2,3]thiadiazole (9a)

^{1}H NMR (300 MHz)

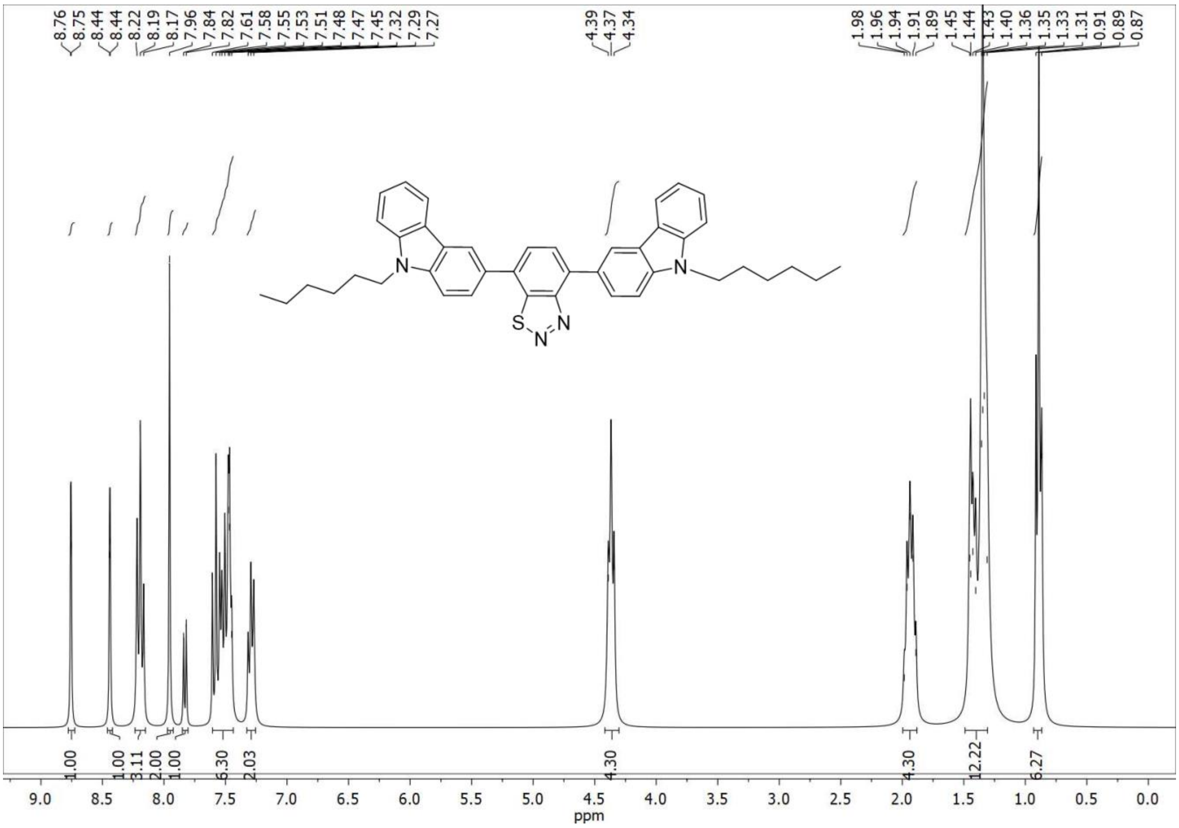

^{13}C NMR (75 MHz)

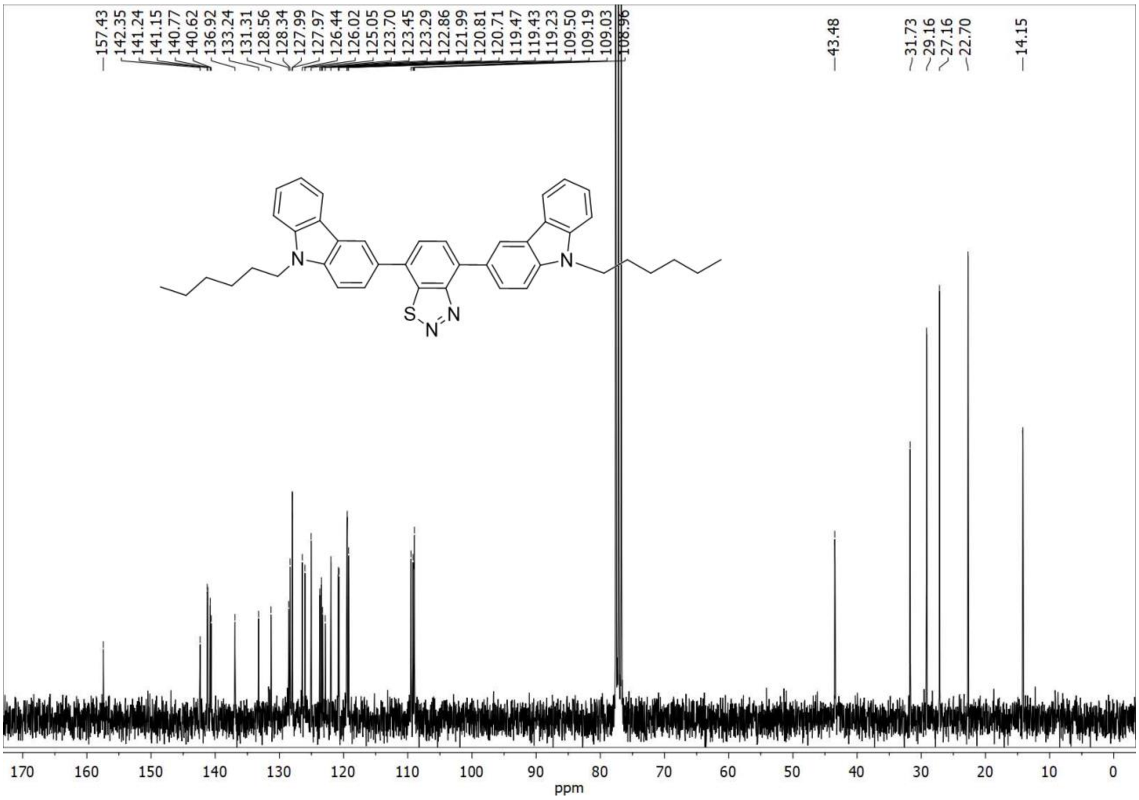

7-Bromo-4-(9-(*p*-tolyl)-2,3,4,4a,9,9a-hexahydro-1*H*-1,4-methanocarbazol-6-yl)benzo[*d*][1,2,3]thiadiazole (7b)

^{1}H NMR (300 MHz)

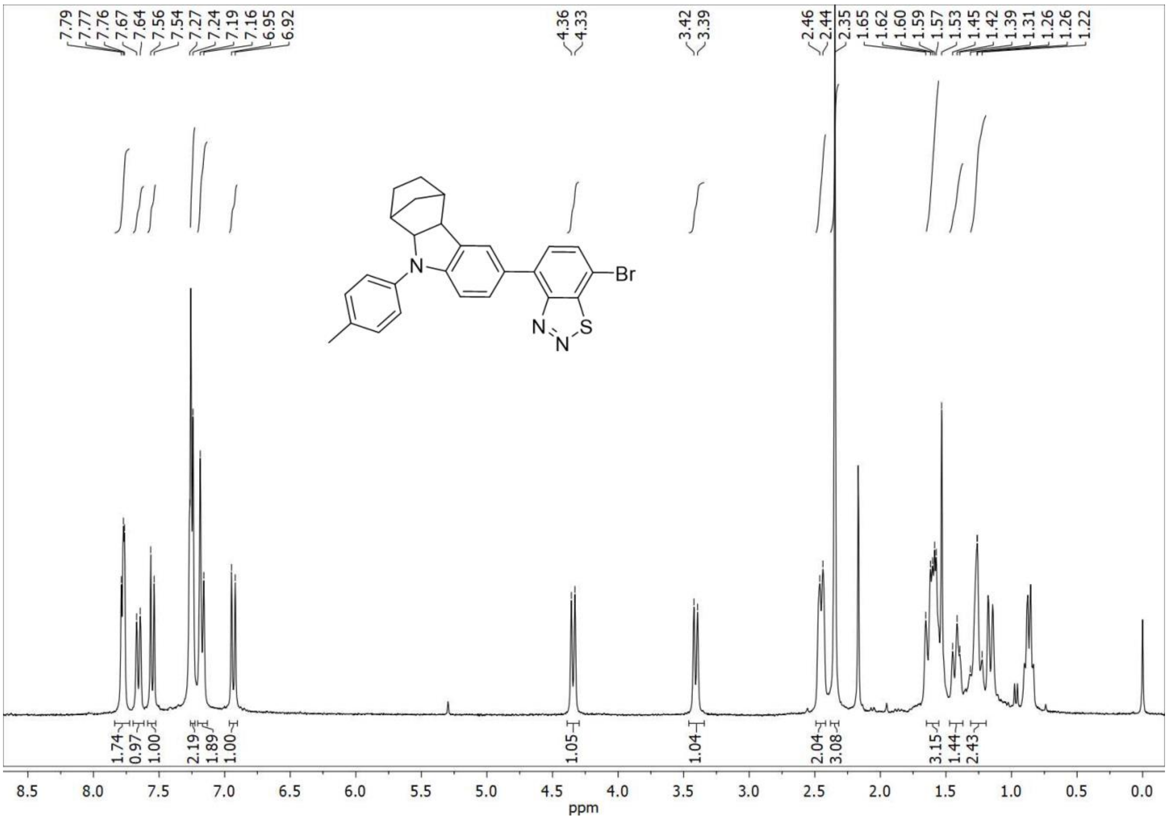

^{13}C NMR (75 MHz)

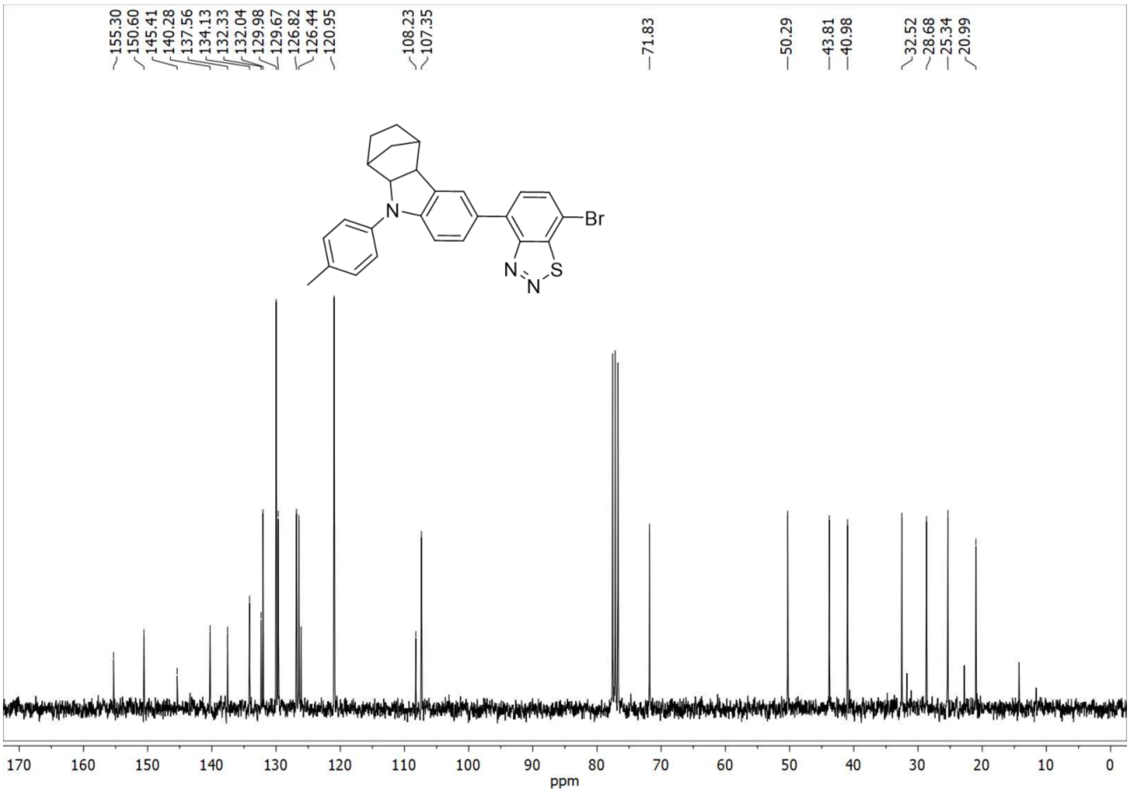

^{1}H,^{1}H-COSY (300MHz)

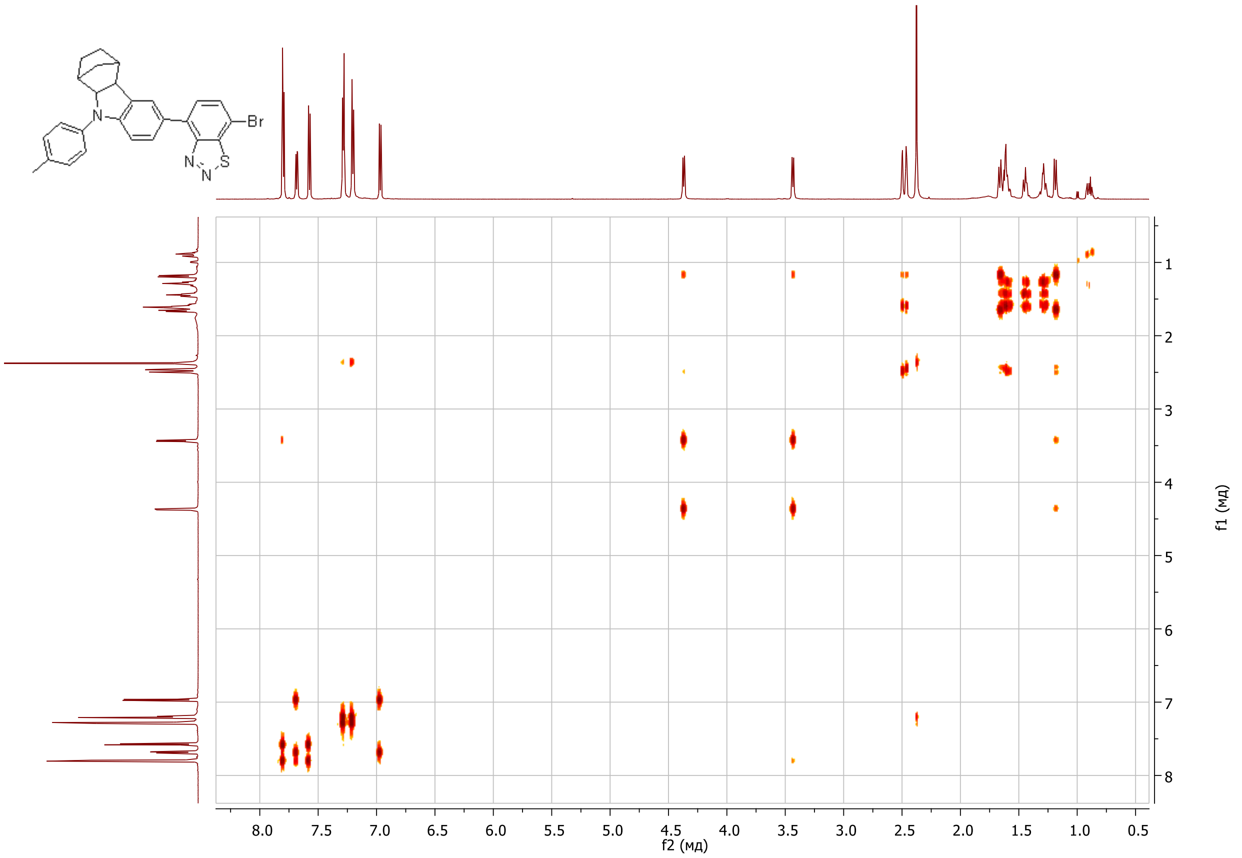

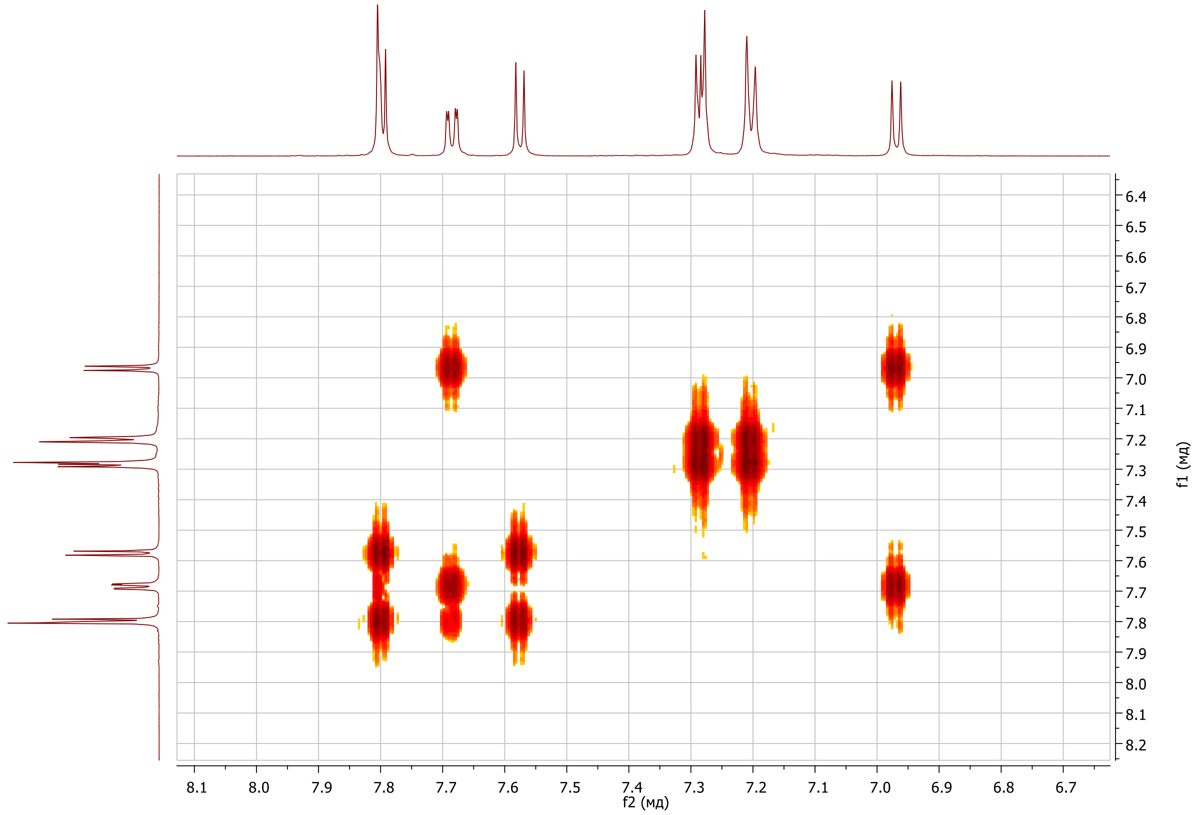

$^{1}H,^{13}C$-HSQC (^{1}H: 300MHz, ^{13}C: 75MHz)

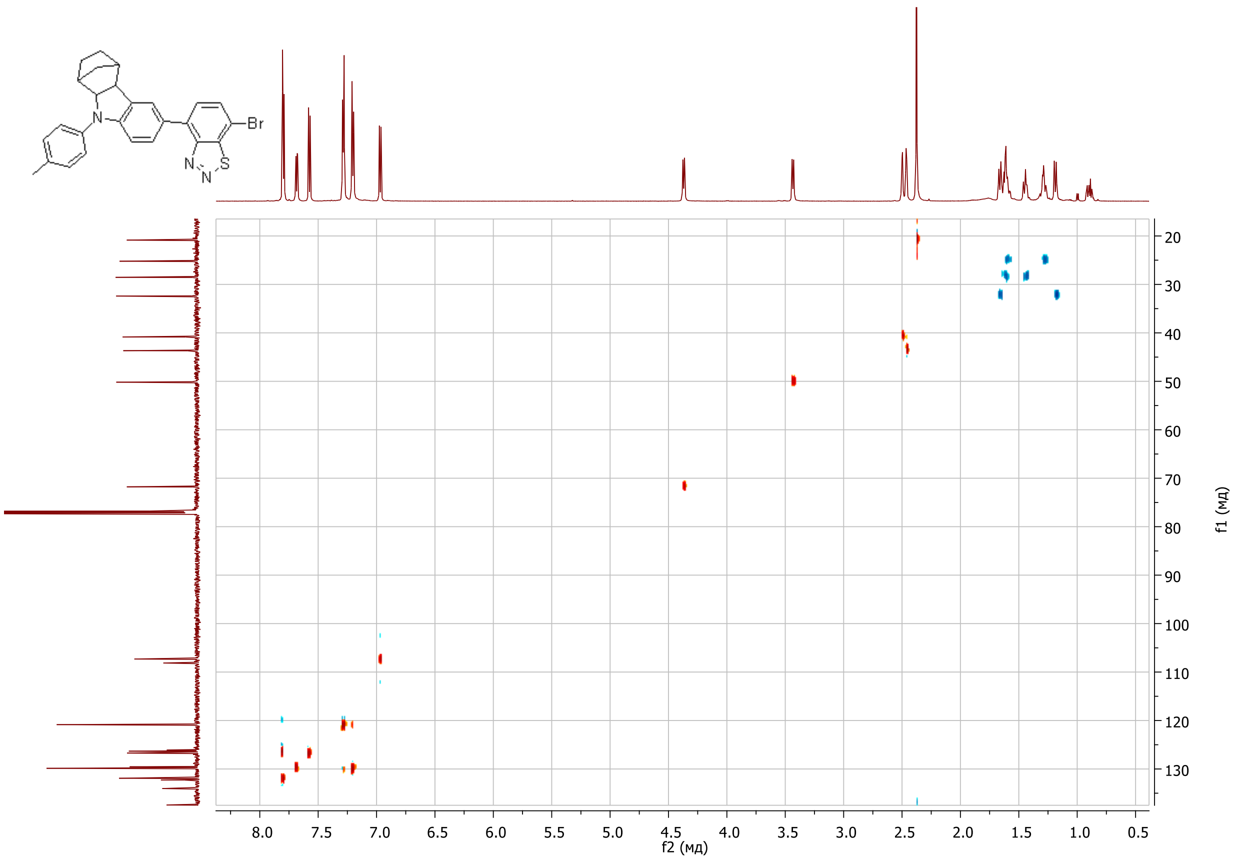

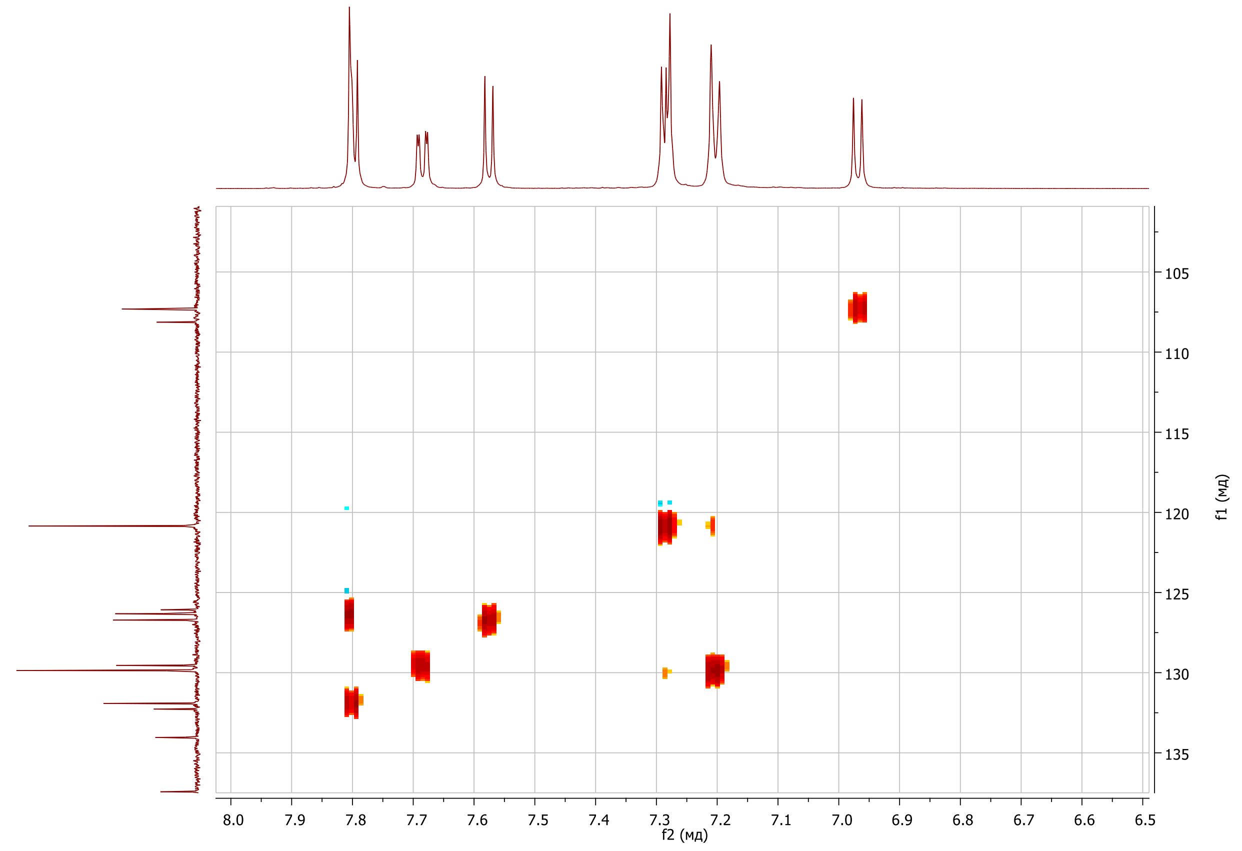

^{1}H,^{13}C-HMBC (^{1}H: 300MHz, ^{13}C: 75MHz)

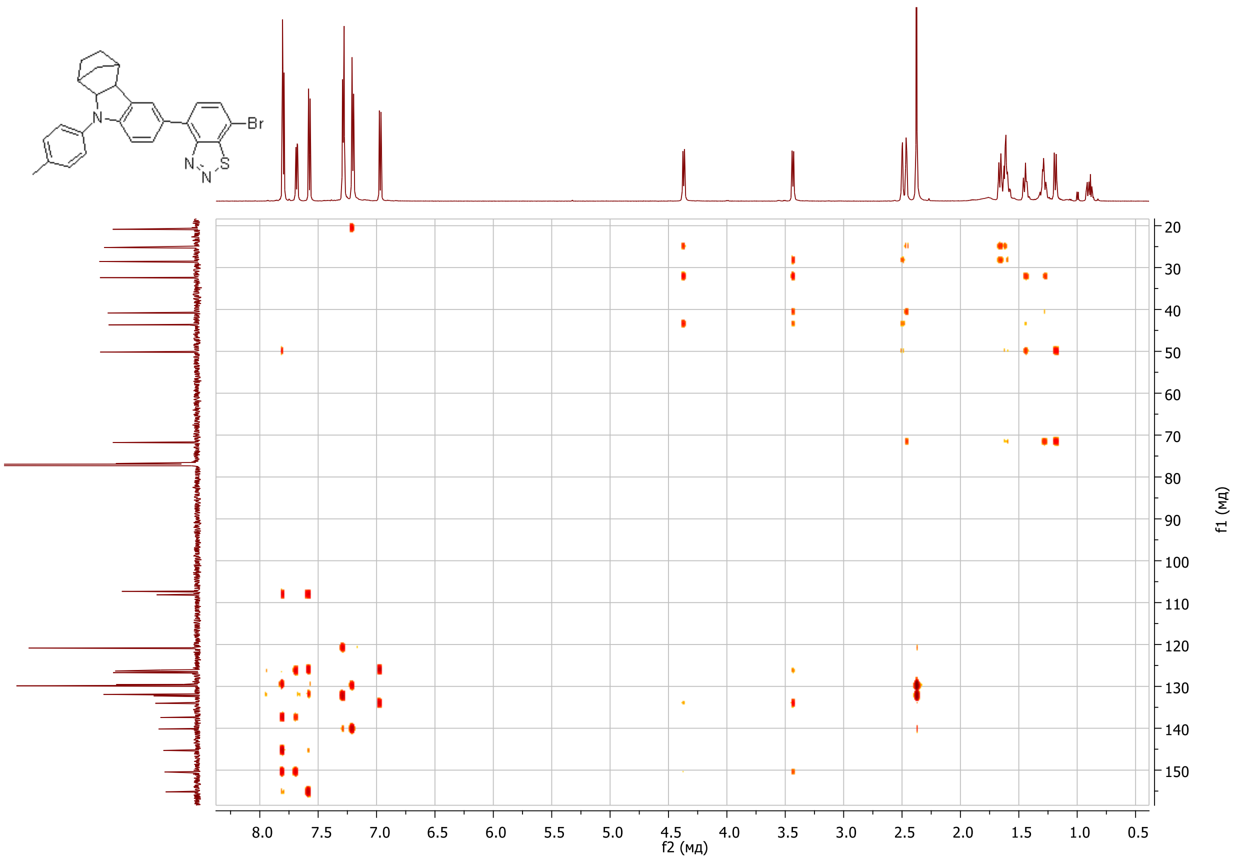

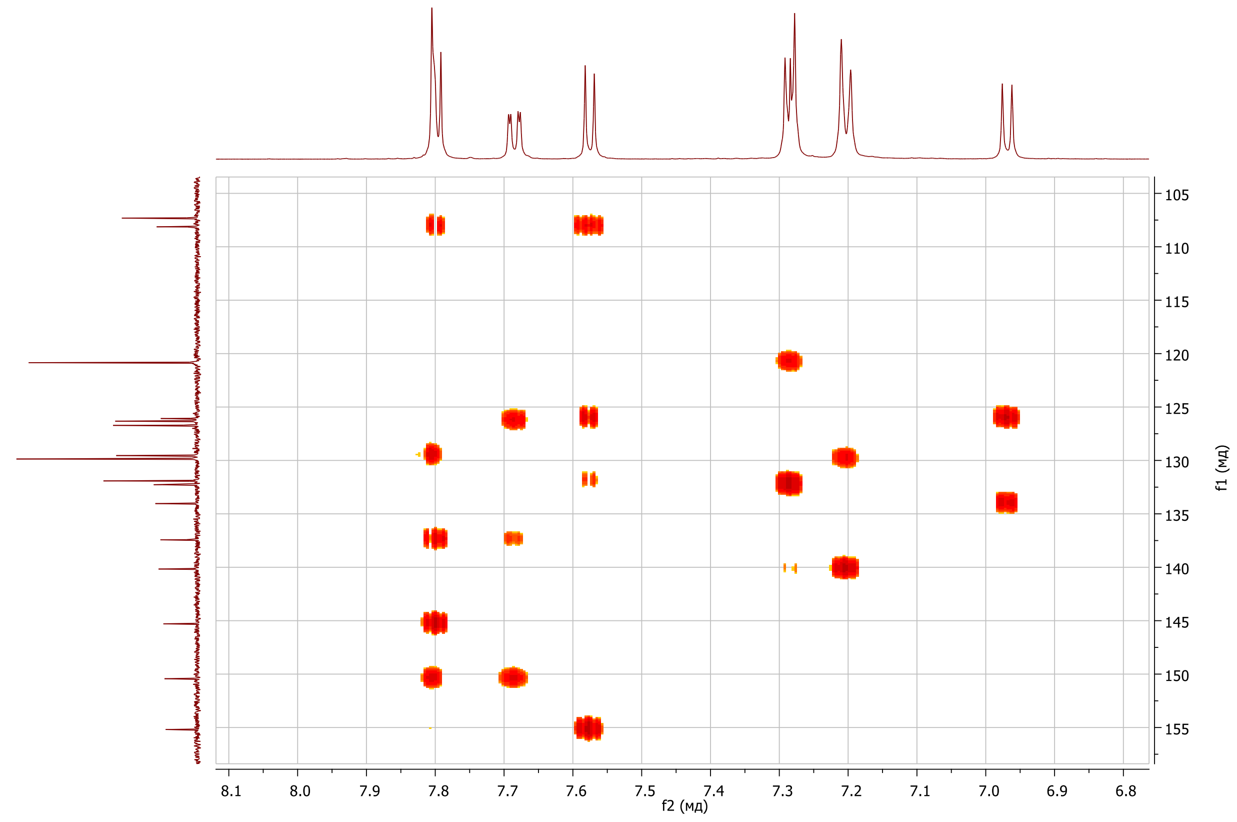

4-Bromo-7-(9-(p-tolyl)-2,3,4,4a,9,9a-hexahydro-1H-1,4-methanocarbazol-6-yl)benzo[d][1,2,3]thiadiazole (8b)

^{1}H NMR (300 MHz)

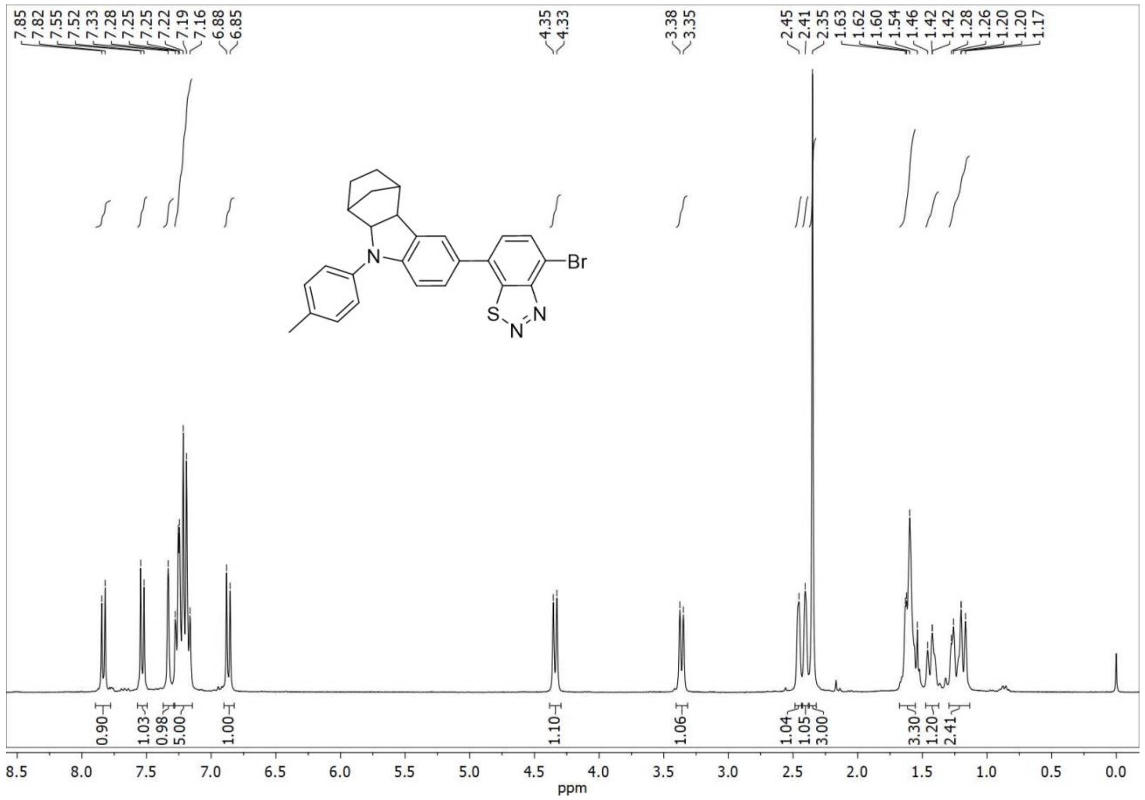

^{13}C NMR (75 MHz)

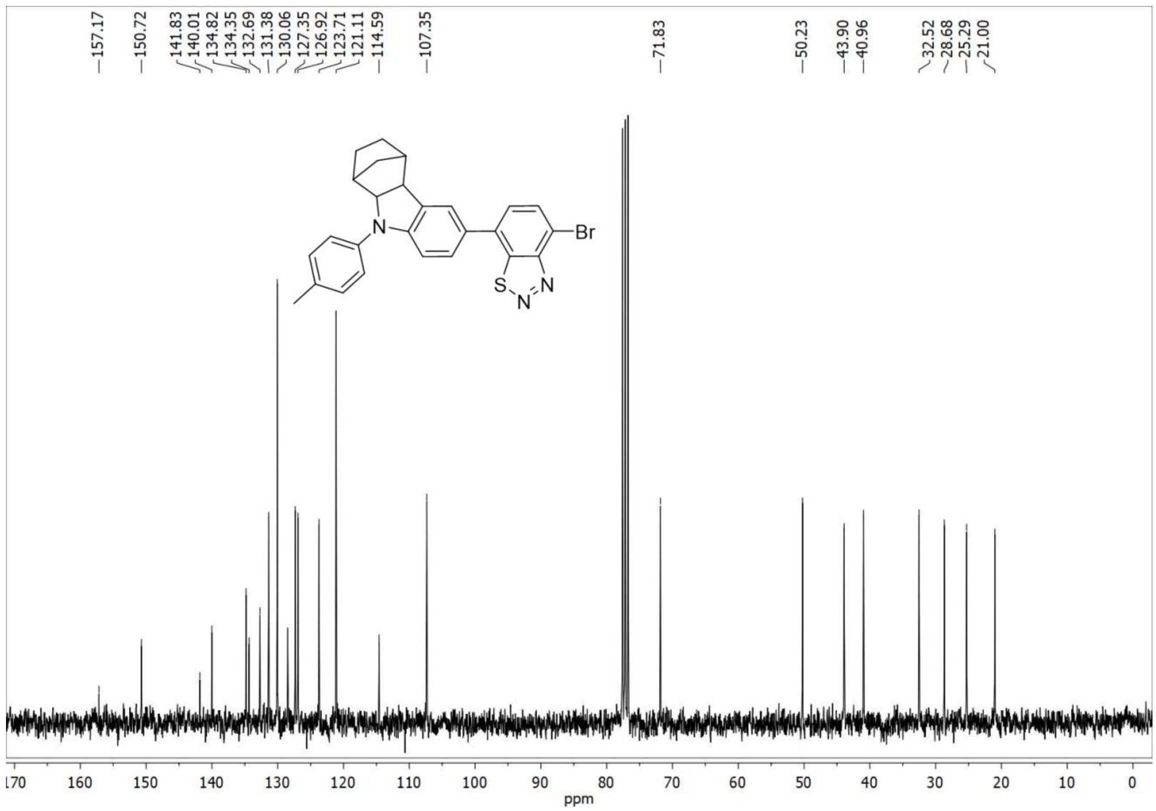

^{1}H,^{1}H-COSY (300MHz)

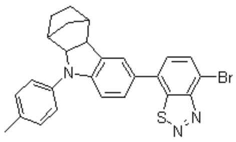

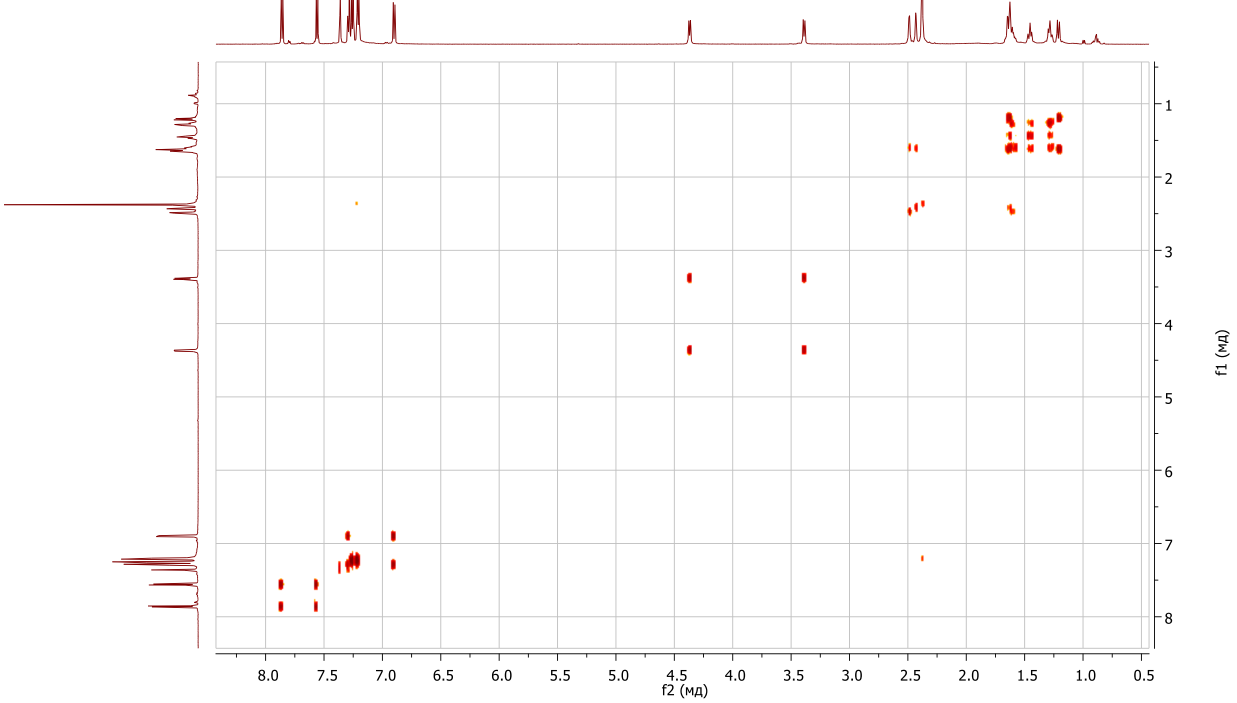

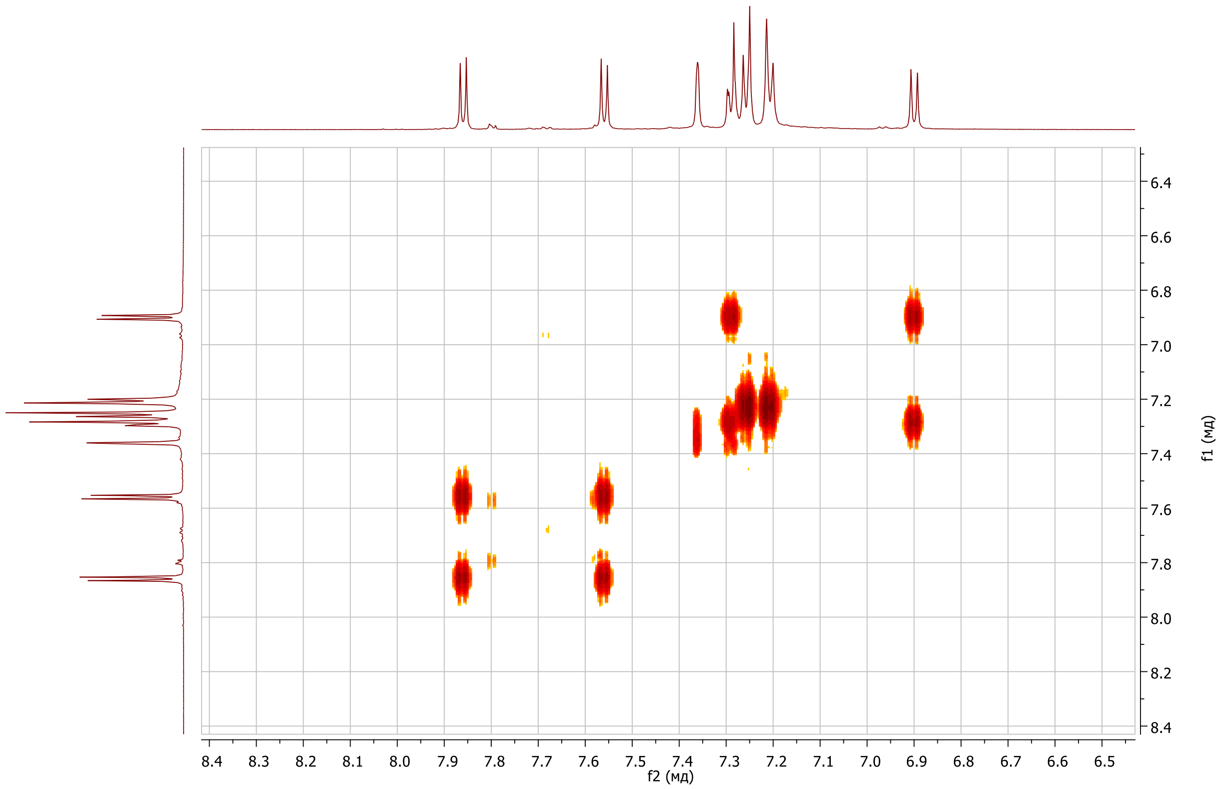

$^{1}H,^{13}C$-HSQC (^{1}H: 300MHz, ^{13}C: 75MHz)

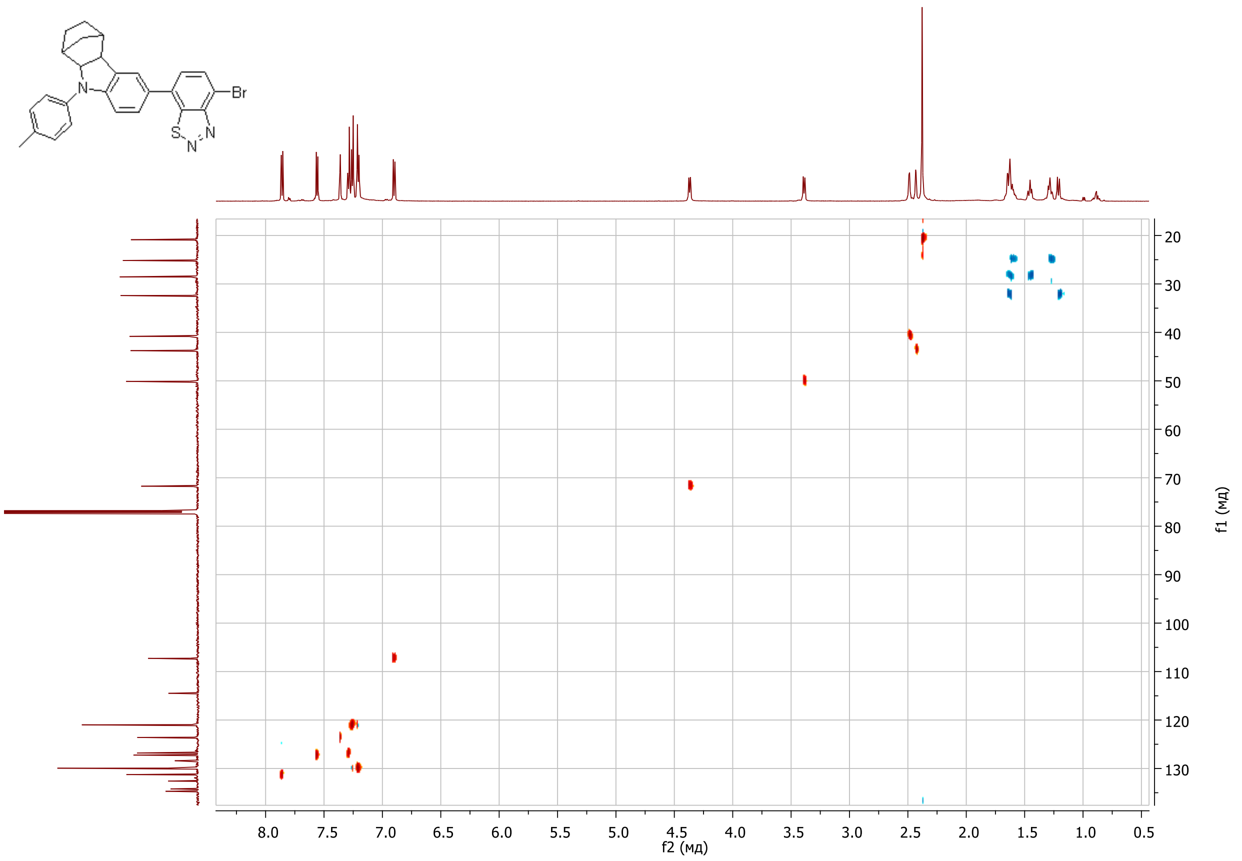

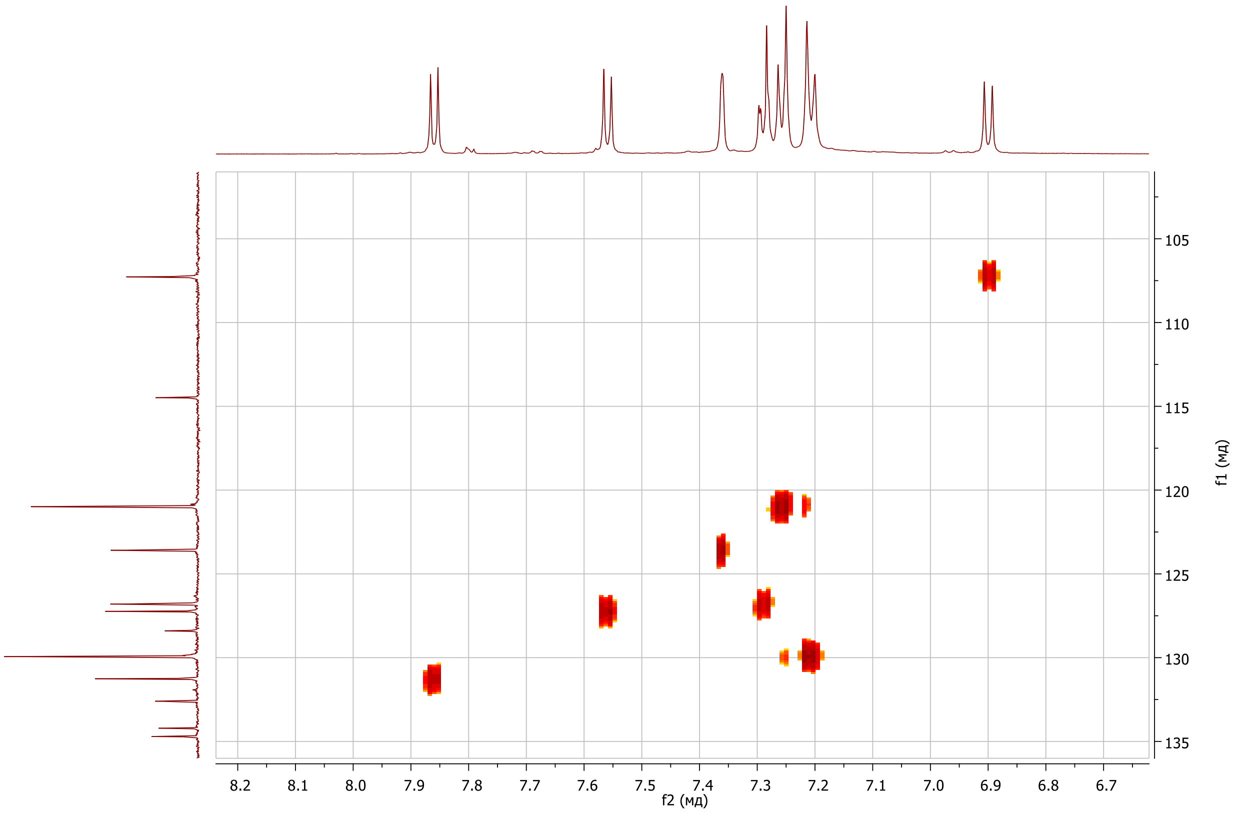

$^{1}H,^{13}C$-HMBC (^{1}H: 300MHz, ^{13}C: 75MHz)

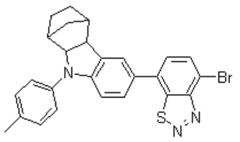

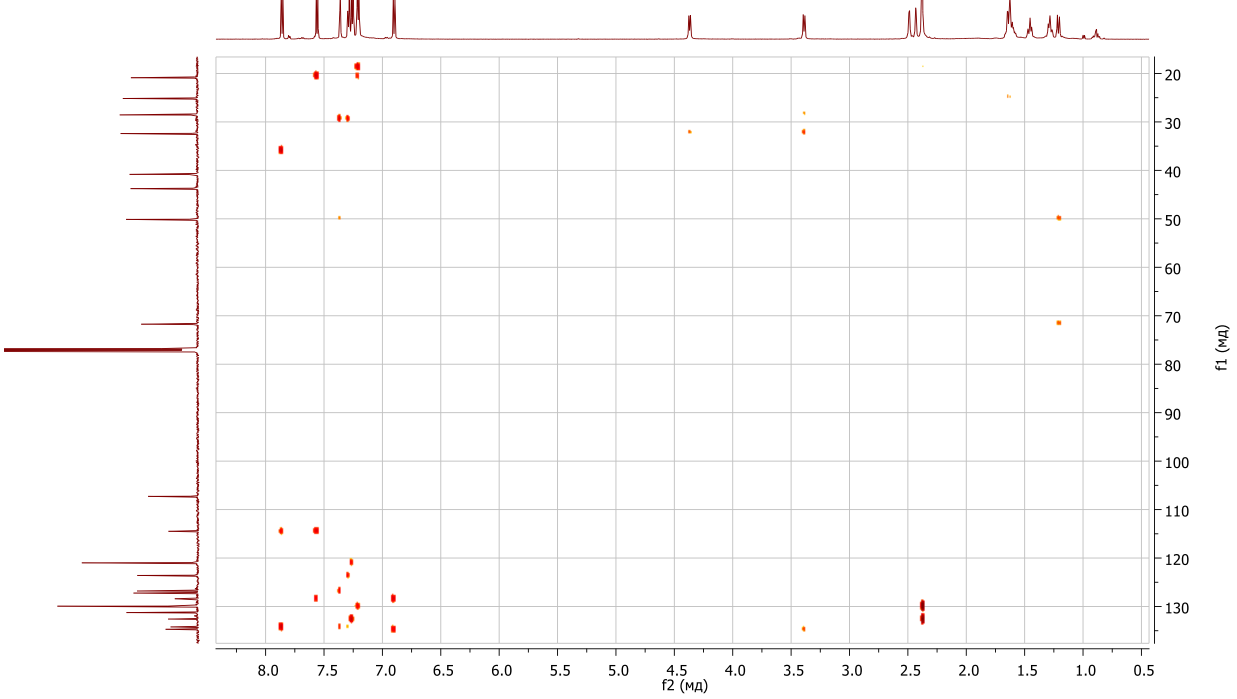

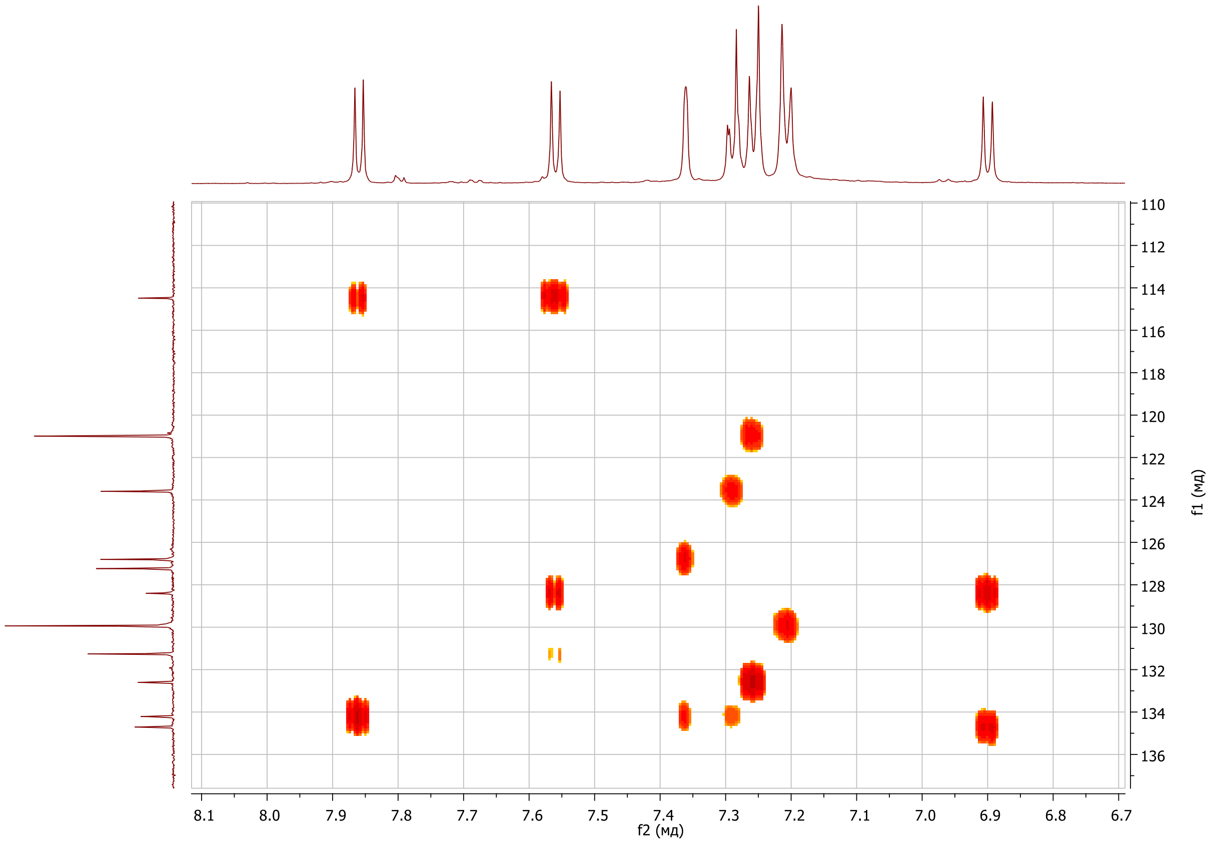

4,7-Bis(9-(*p*-tolyl)-2,3,4,4a,9,9a-hexahydro-1*H*-1,4-methanocarbazol-6-yl)benzo[*d*][1,2,3]thiadiazole (9b)

^{1}H NMR (300 MHz)

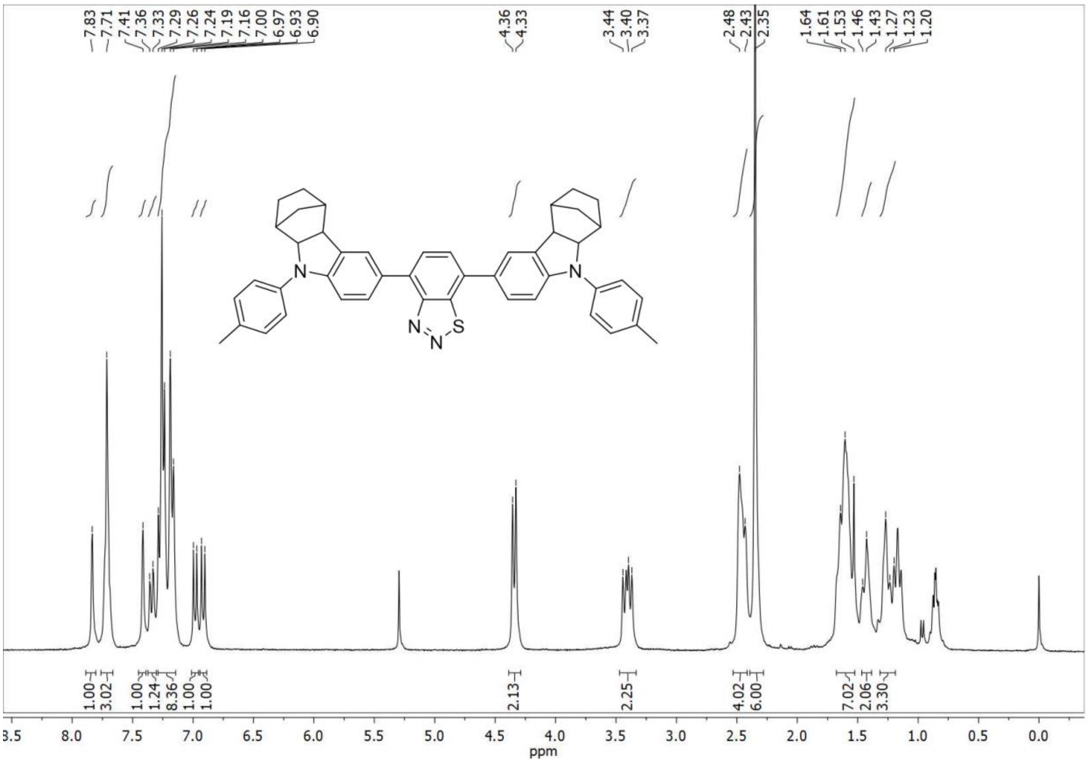

^{13}C NMR (75 MHz)

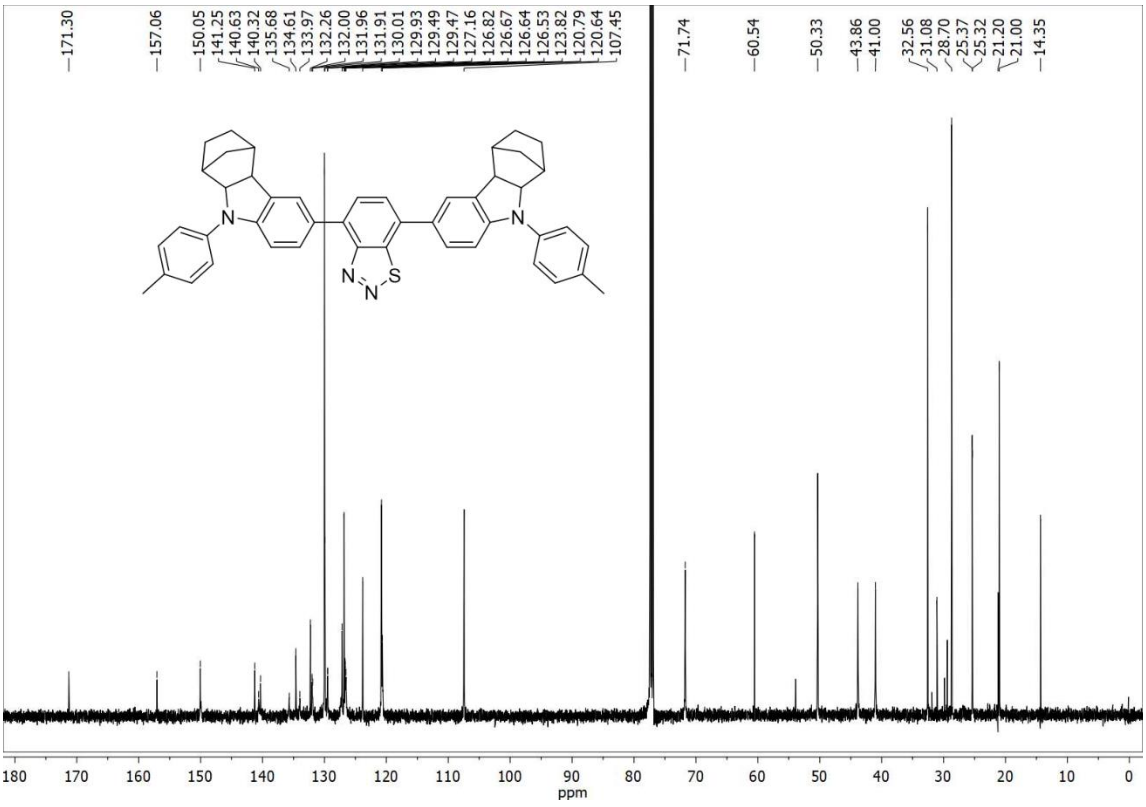

7-Bromo-4-(9-(*p*-tolyl)-2,3,4,4a,9,9a-hexahydro-1*H*-carbazole-6-yl)benzo[*d*][1,2,3]thiadiazole (7c)

^{1}H NMR (300 MHz)

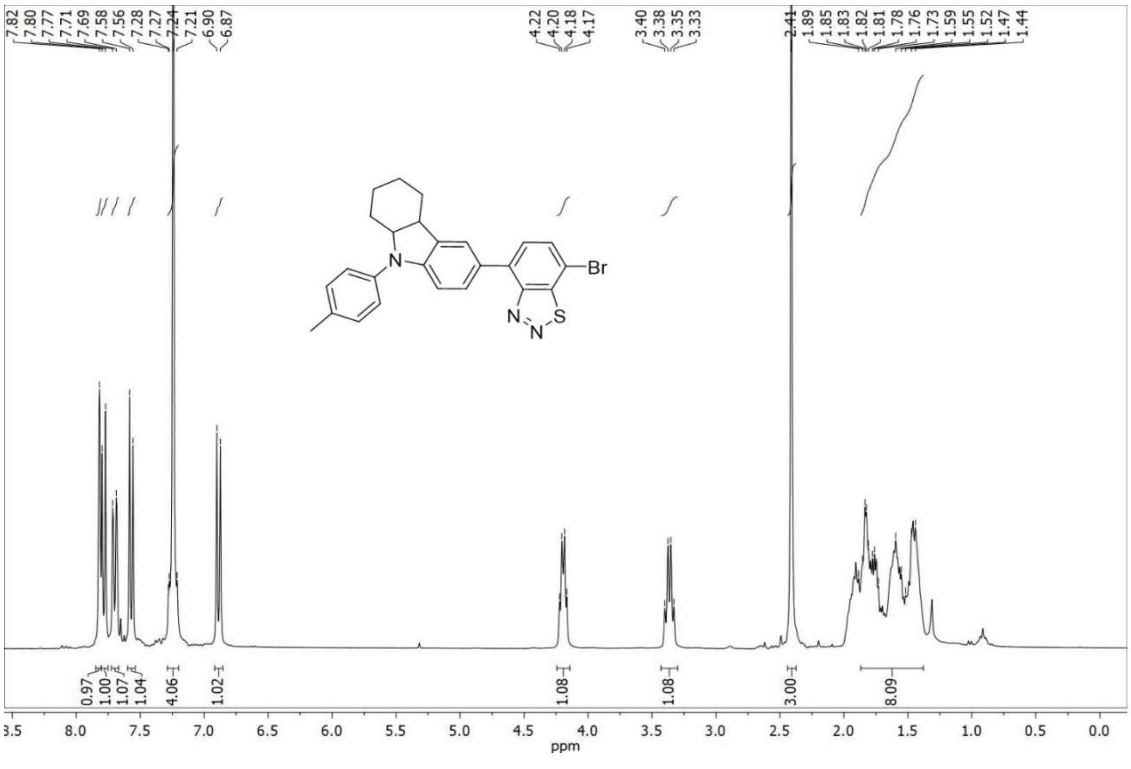

^{13}C NMR (75 MHz)

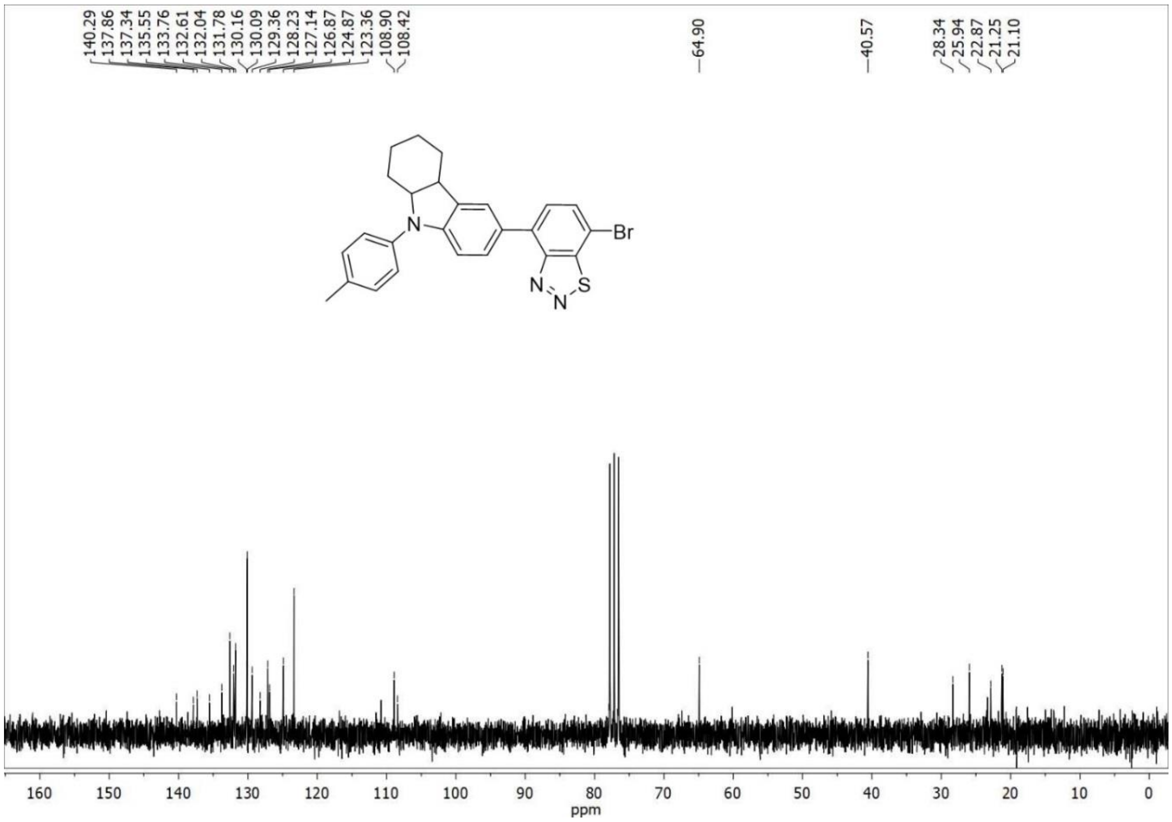

4-Bromo-7-(9-(*p*-tolyl)-2,3,4,4a,9,9a-hexahydro-1*H*-carbazole-6-yl)benzo[*d*][1,2,3]thiadiazole (8c)

^{1}H NMR (300 MHz)

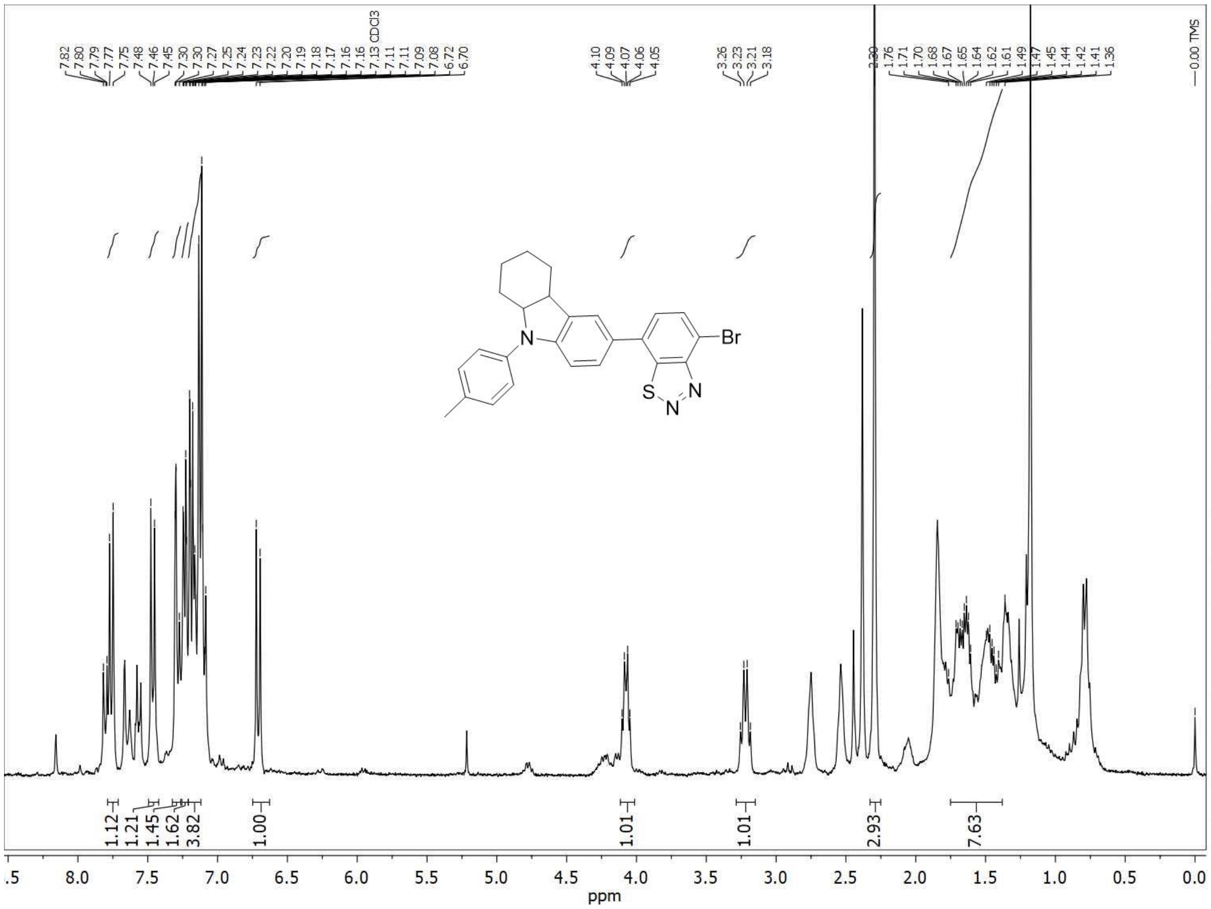

MALDI-TOF

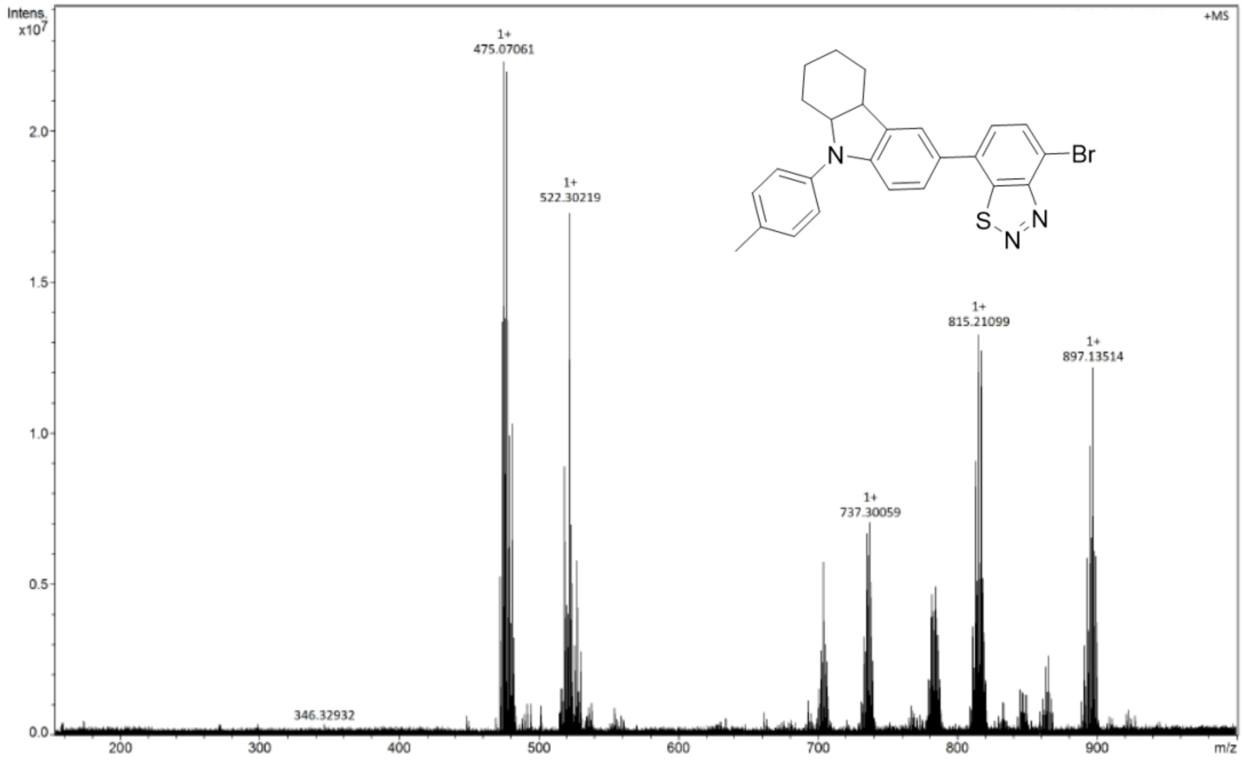

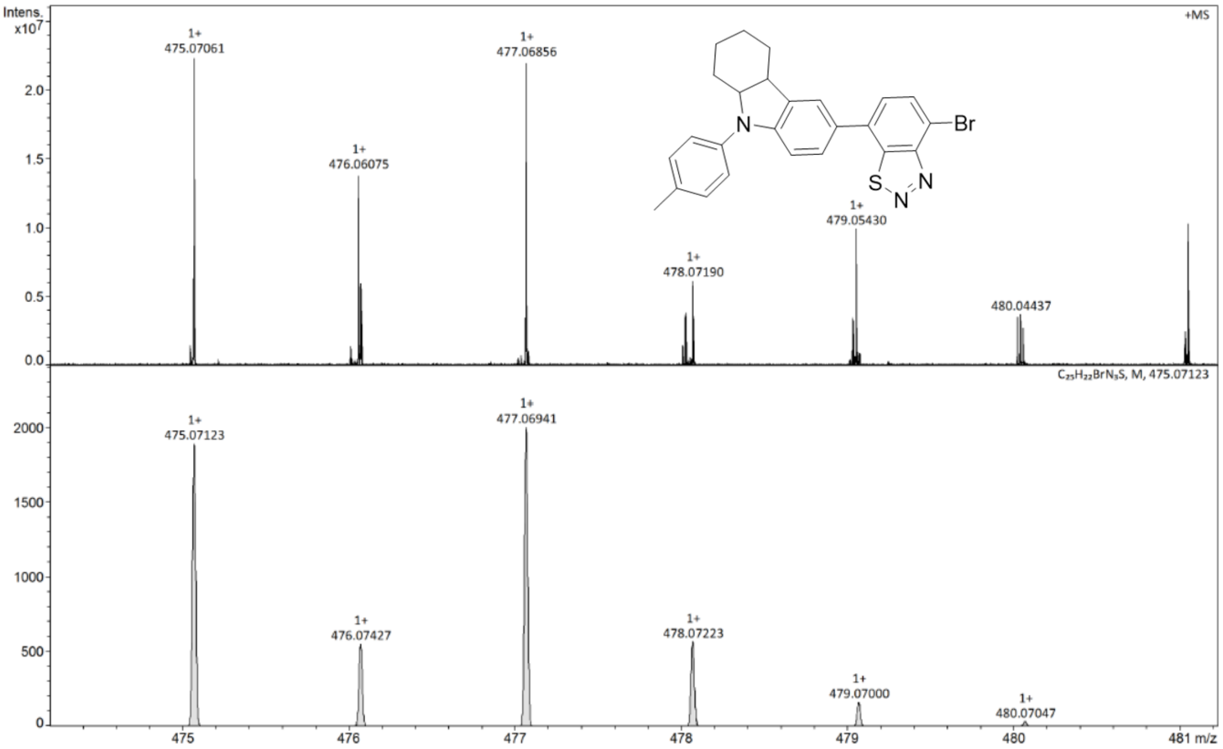

Intens.
x10^7
+MS
475.07061
476.06075
477.06856
478.07190
479.05430
480.04437
N
Br
S
N
N
C₂₅H₂₂BrN₃S, M, 475.07123
475.07123
476.07427
477.06941
478.07223
479.07000
480.07047
m/z

4,7-Bis(9-(*p*-tolyl)-2,3,4,4a,9,9a-hexahydro-1*H*-carbazole-6-yl)benzo[*d*][1,2,3]thiadiazole (9c)

1H NMR (300 MHz)

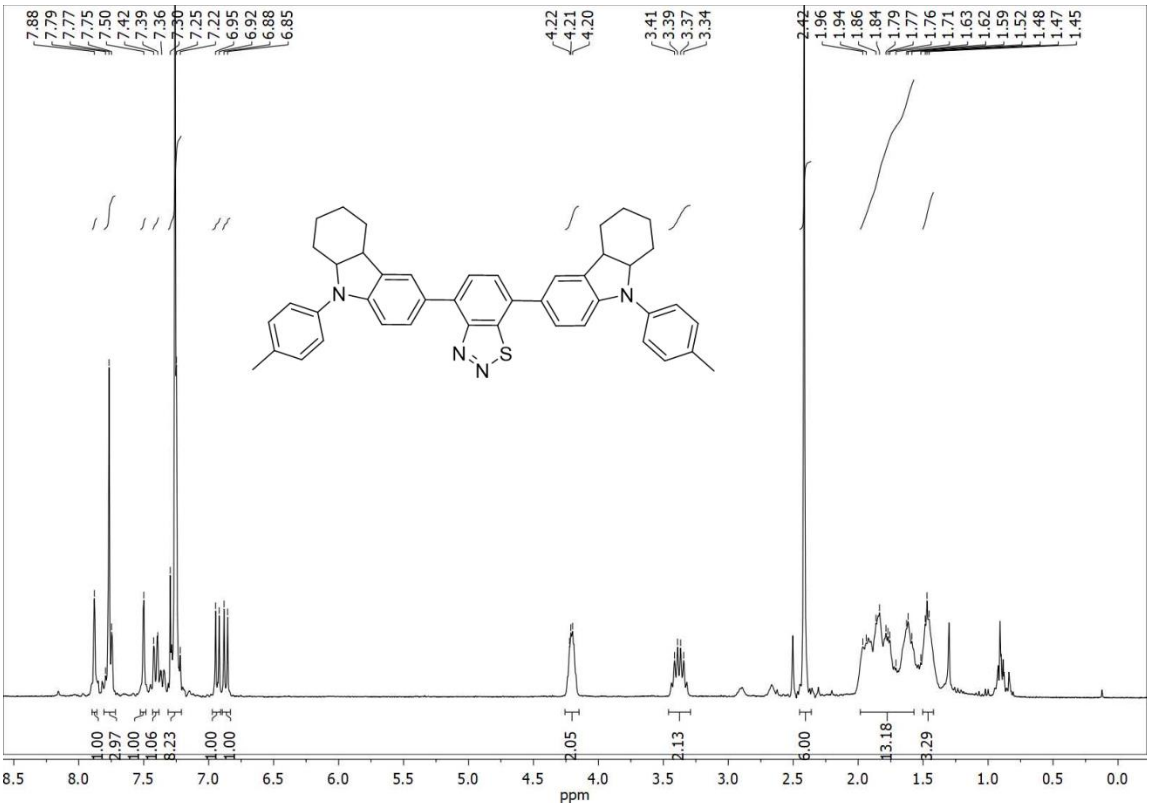

13C NMR (75 MHz)

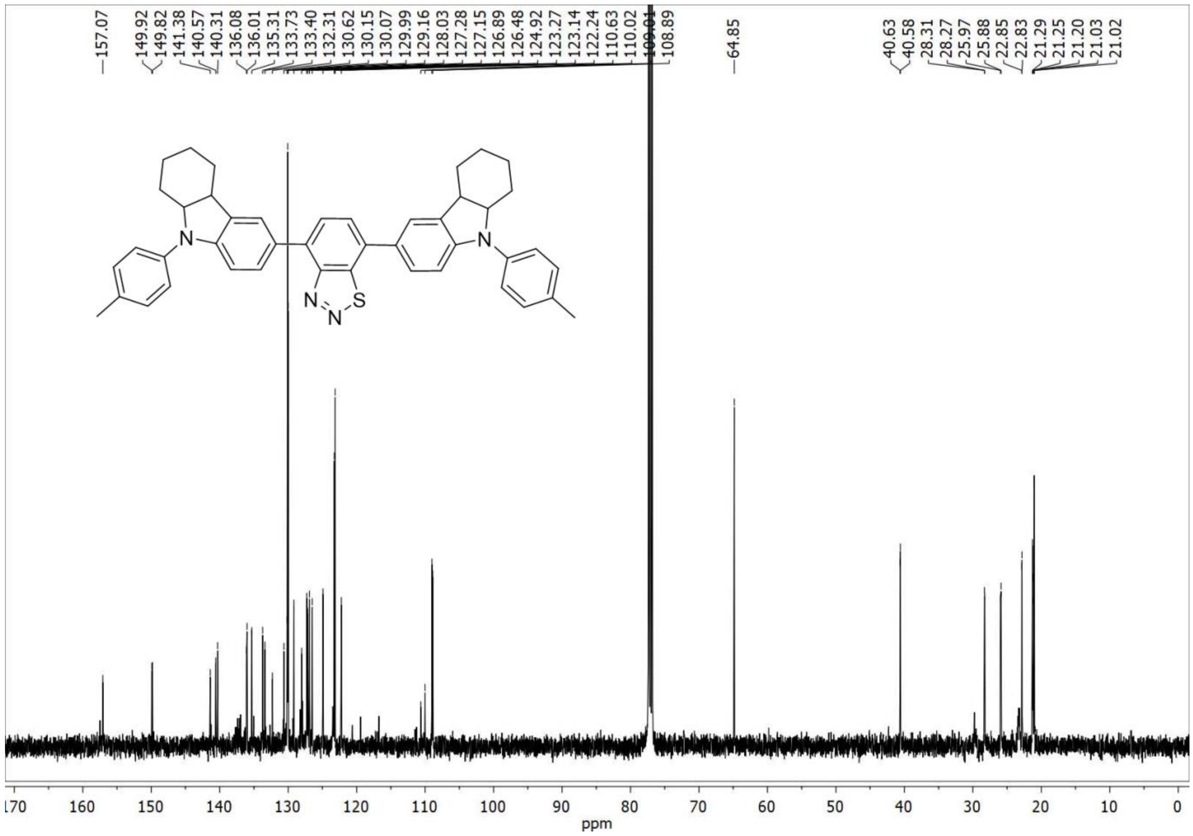

7-Bromo-4-(4-(*p*-tolyl)-1,2,3,3a,4,8b-hexahydrocyclopenta[*b*]indol-7-yl)benzo[*d*][1,2,3]thiadiazole (7d)

^{1}H NMR (300 MHz)

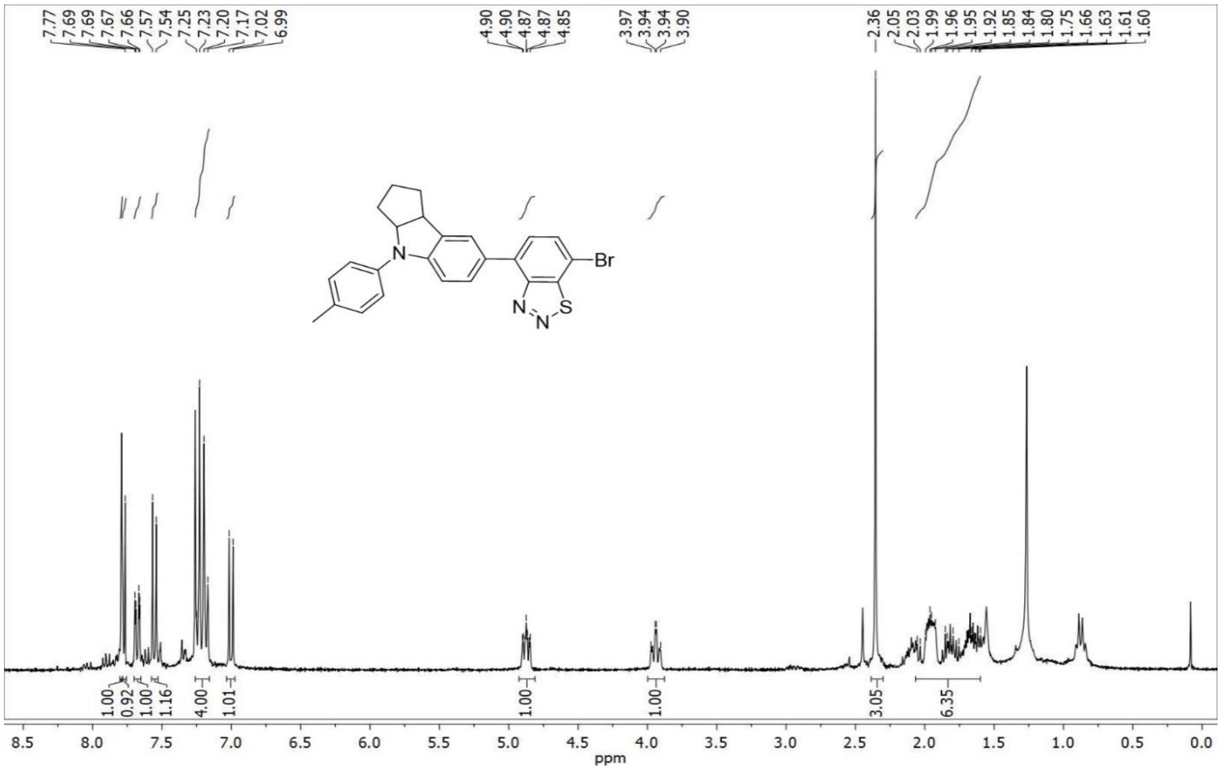

^{13}C NMR (75 MHz)

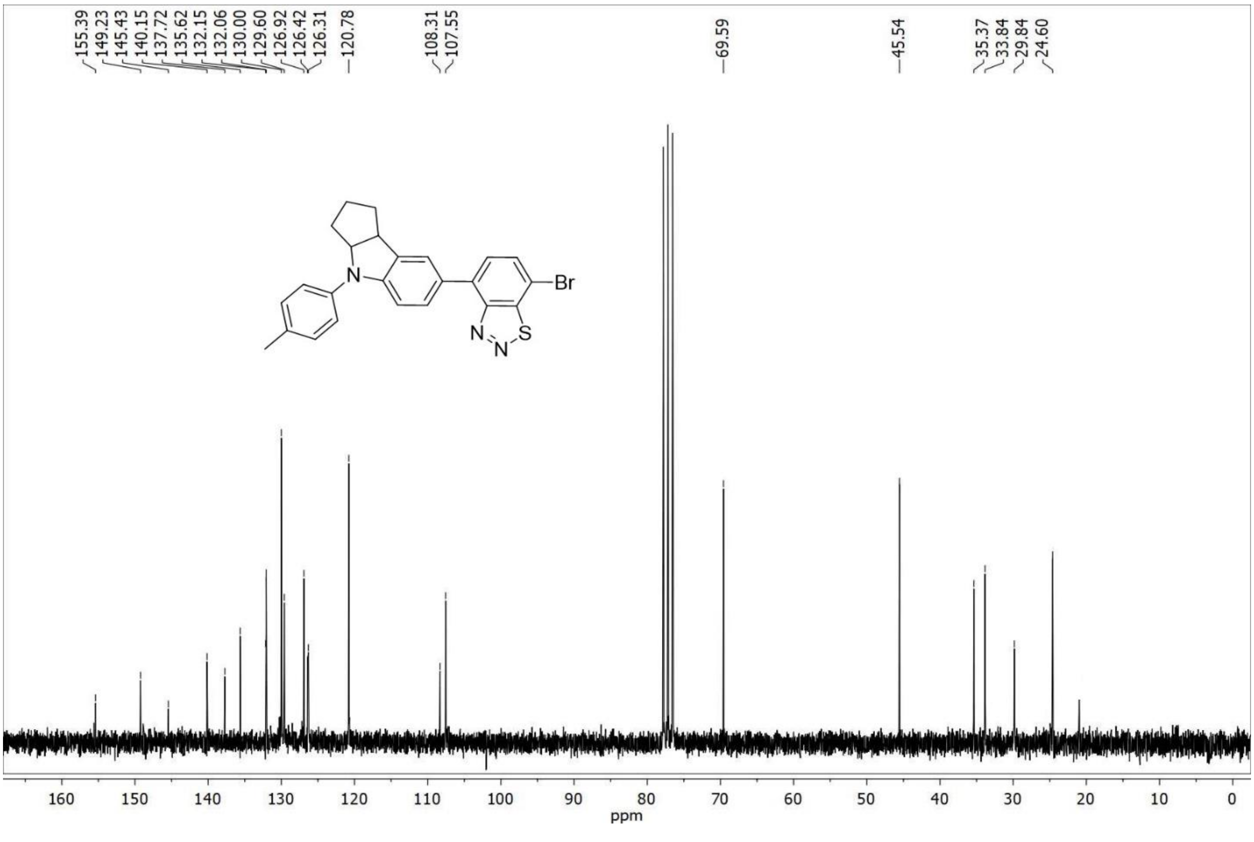

4-Bromo-7-(4-(*p*-tolyl)-1,2,3,3a,4,8b-hexahydrocyclopenta[*b*]indol-7-yl)benzo[*d*][1,2,3]thiadiazole (8d)

^{1}H NMR (300 MHz)

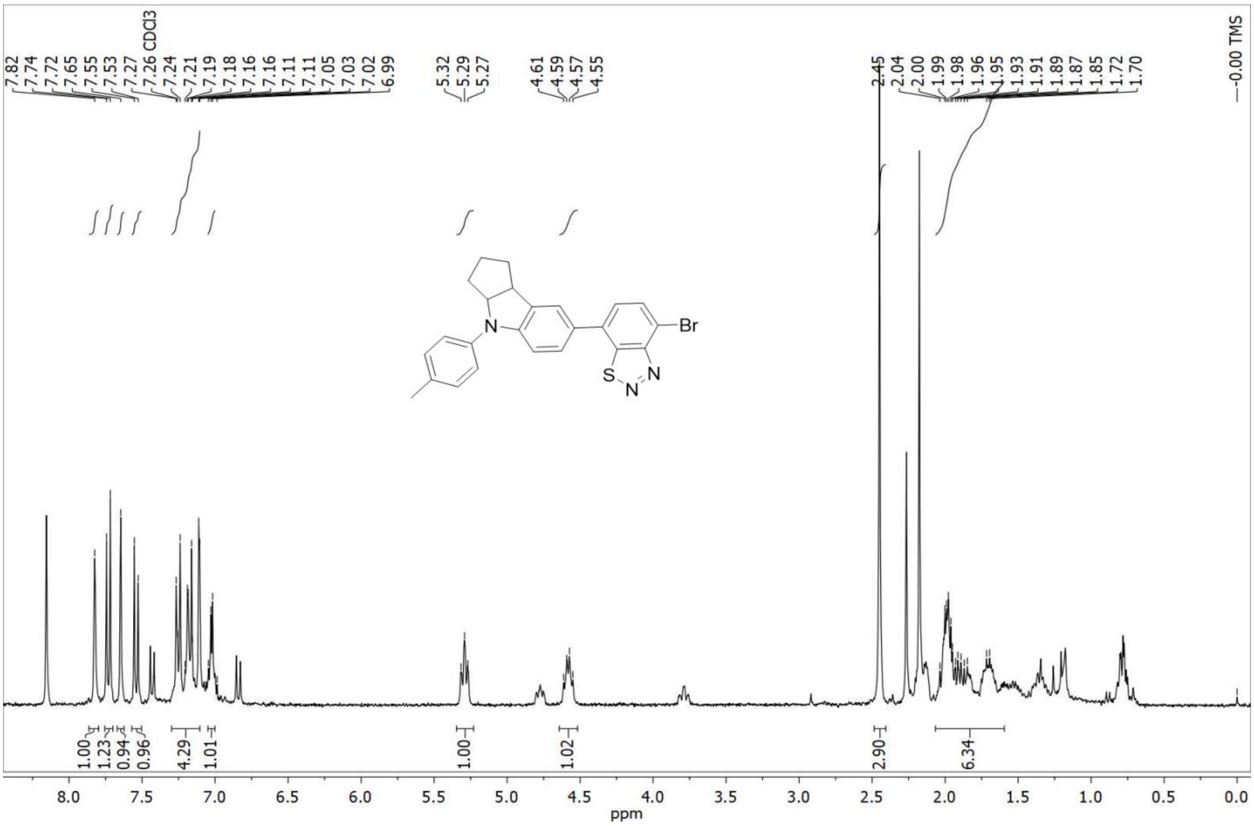

MALDI-TOF

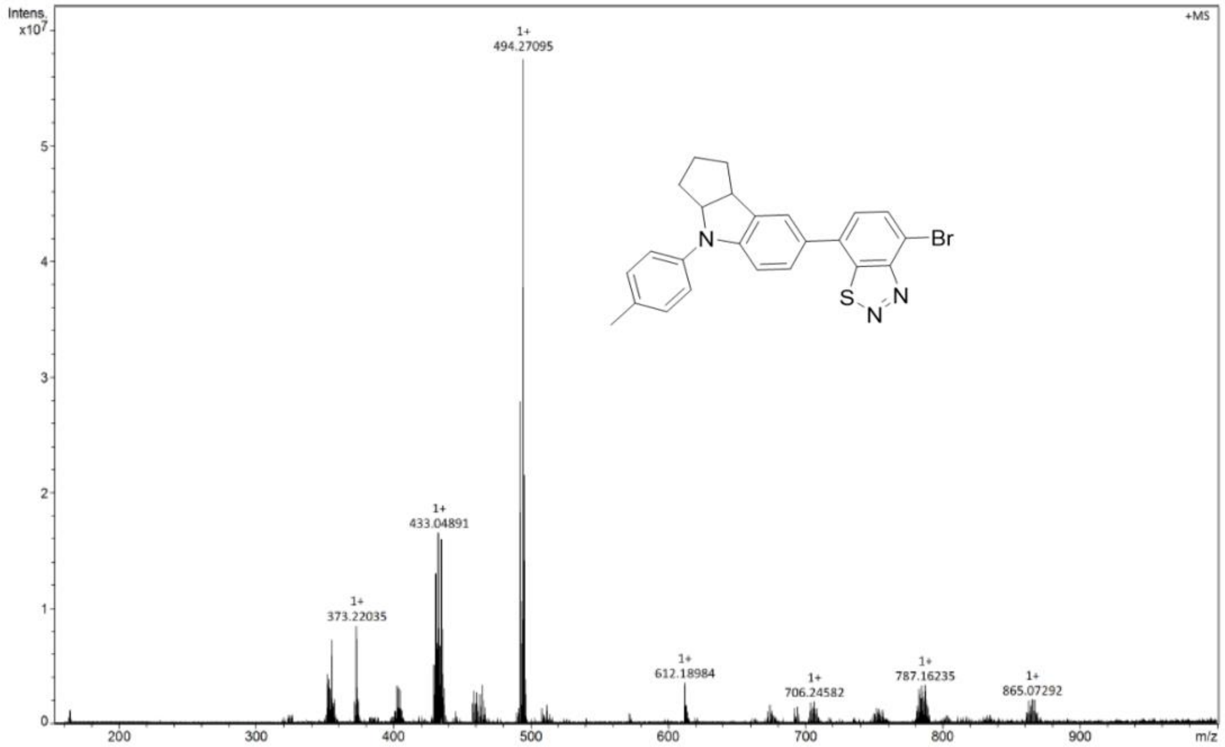

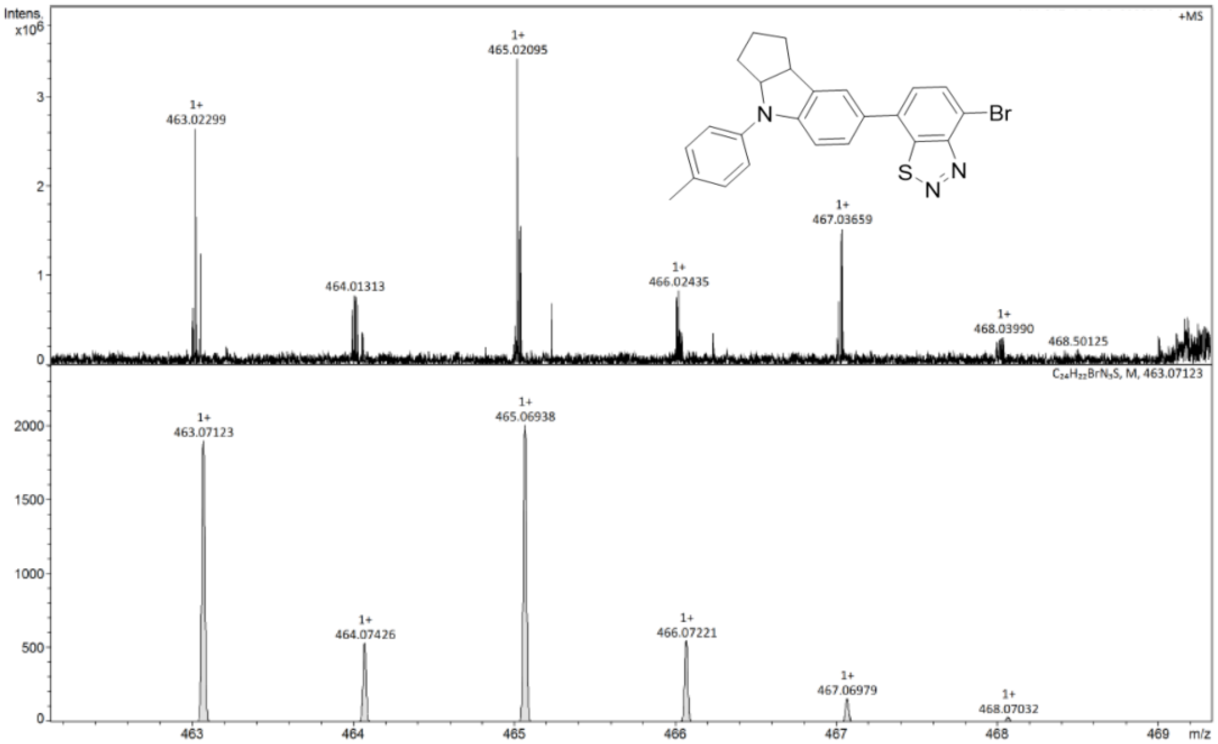
Intens.
x10^6
+MS
1+
463.02299
464.01313
1+
465.02095
1+
466.02435
1+
467.03659
1+
468.03990
468.50125
C24H22BrN3S, M, 463.07123
1+
463.07123
1+
464.07426
1+
465.06938
1+
466.07221
1+
467.06979
1+
468.07032
m/z
Br
N
S
N
N

4,7-Bis(4-(*p*-tolyl)-1,2,3,3a,4,8b-hexahydrocyclopenta[*b*]indol-7-yl)benzo[*d*][1,2,3]thiadiazole (9d)

^{1}H NMR (300 MHz)

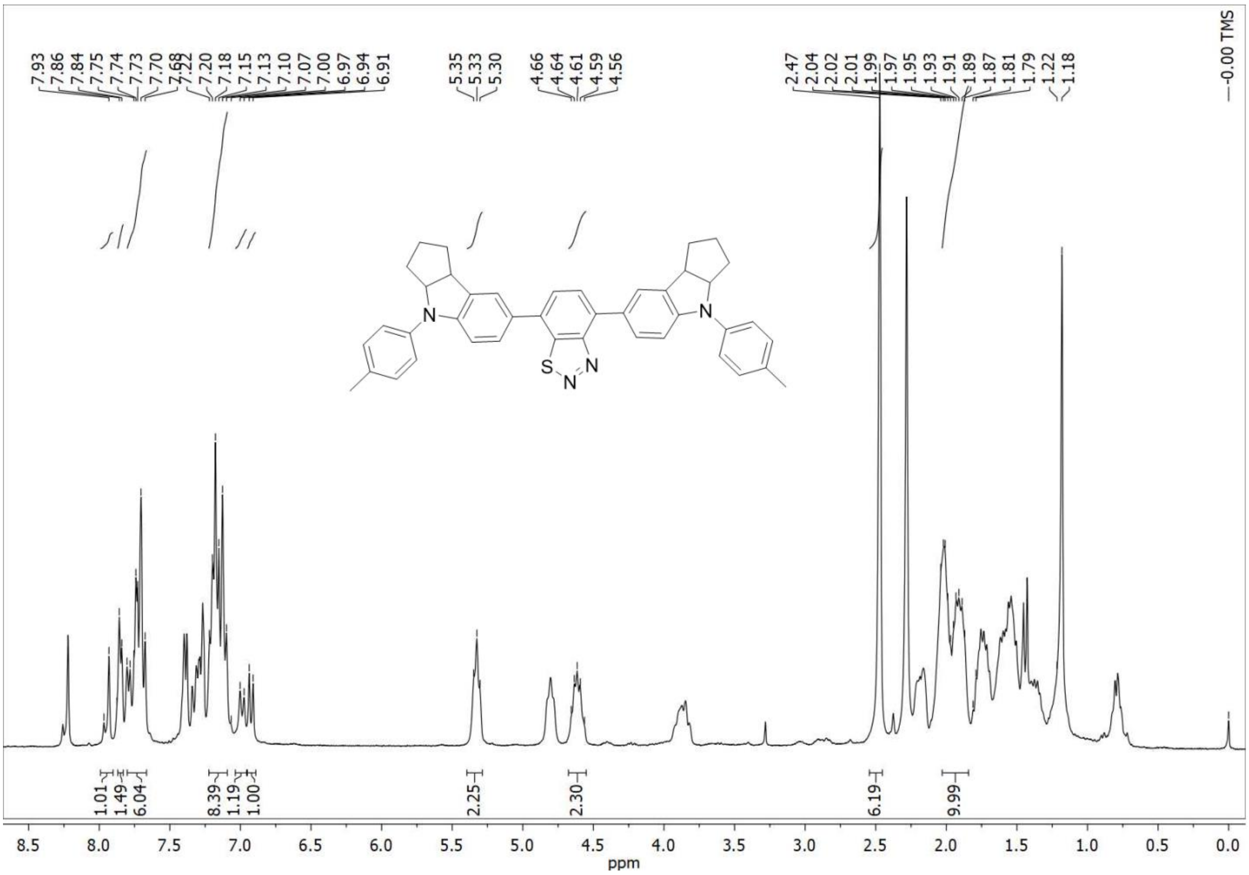

MALDI-TOF

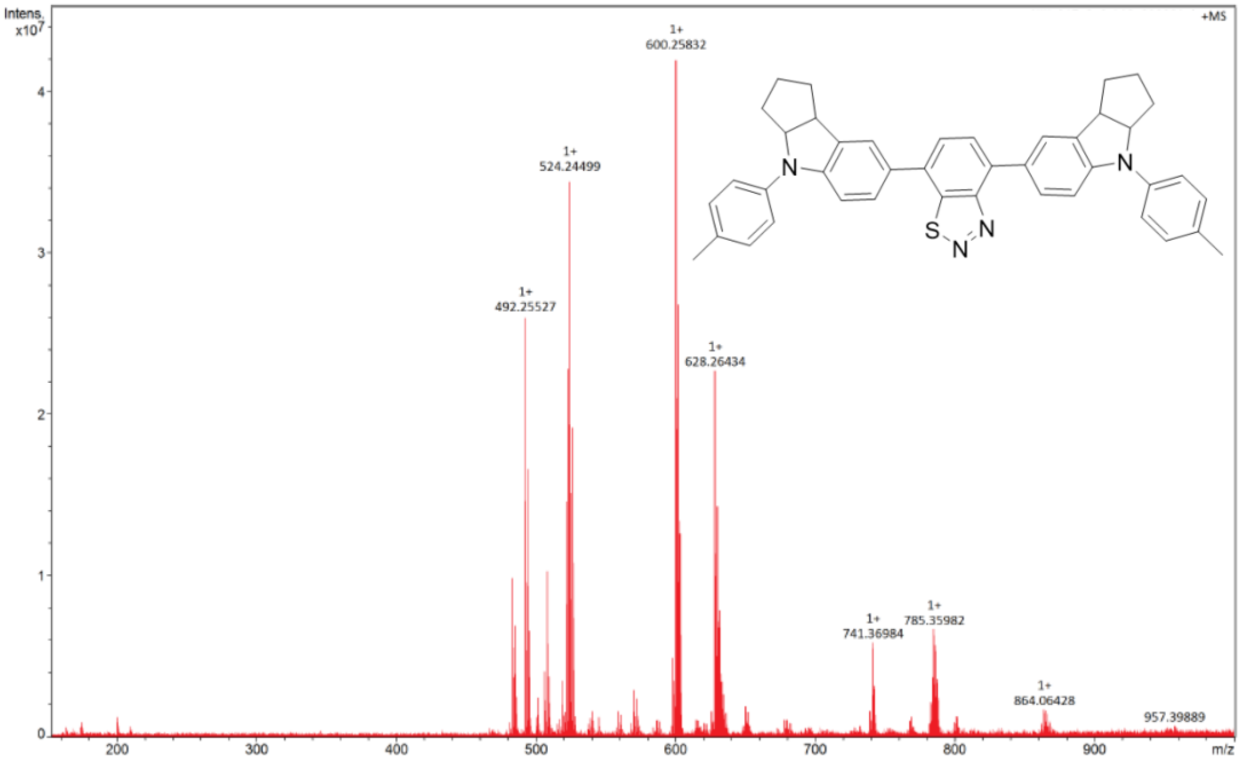

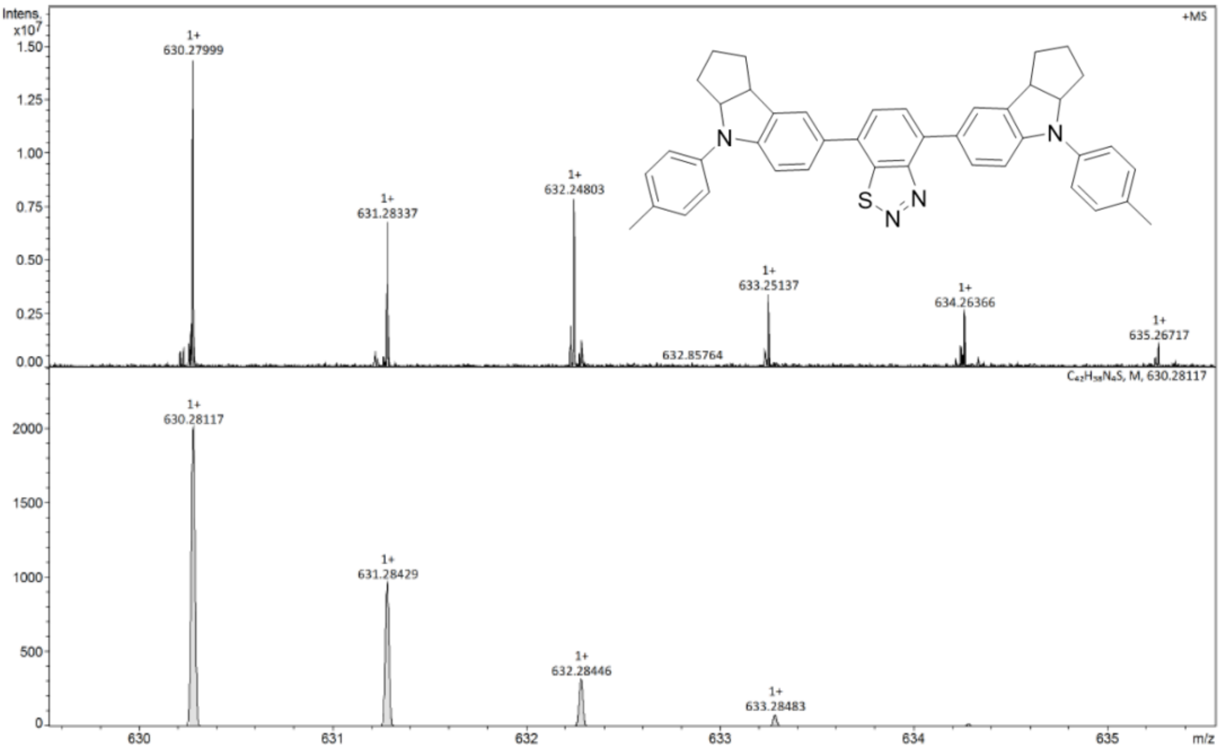
Intens.
x10^7
+MS
1.50
1.25
1.00
0.75
0.50
0.25
0.00
1+
630.27999
1+
631.28337
1+
632.24803
632.85764
1+
633.25137
1+
634.26366
1+
635.26717
N
S
N
N
N
C42H38N4S, M, 630.28117
2000
1500
1000
500
0
1+
630.28117
1+
631.28429
1+
632.28446
1+
633.28483
630
631
632
633
634
635
m/z

7-Bromo-4-phenylbenzo[*d*][1,2,3]thiadiazole (7e)

^{1}H NMR (300 MHz)

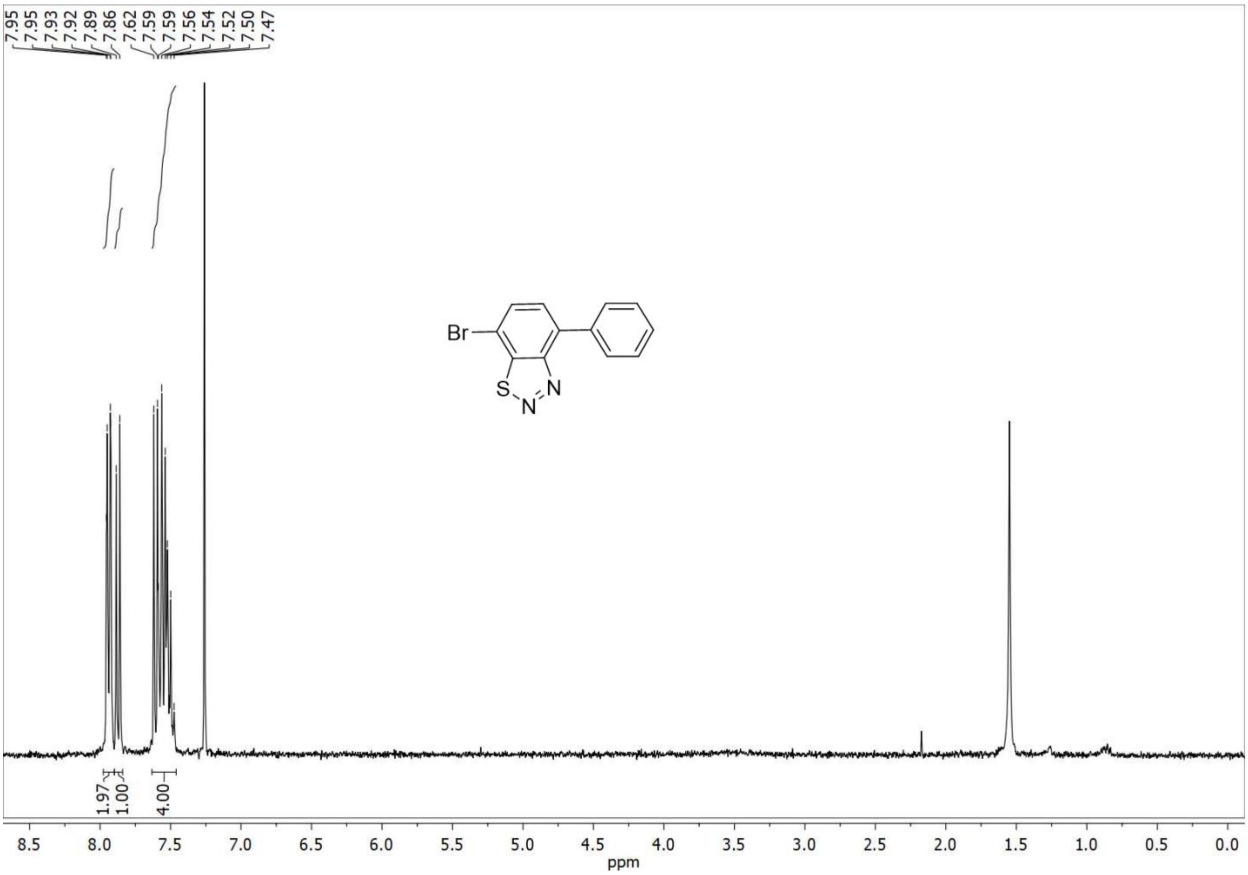

^{13}C NMR (75 MHz)

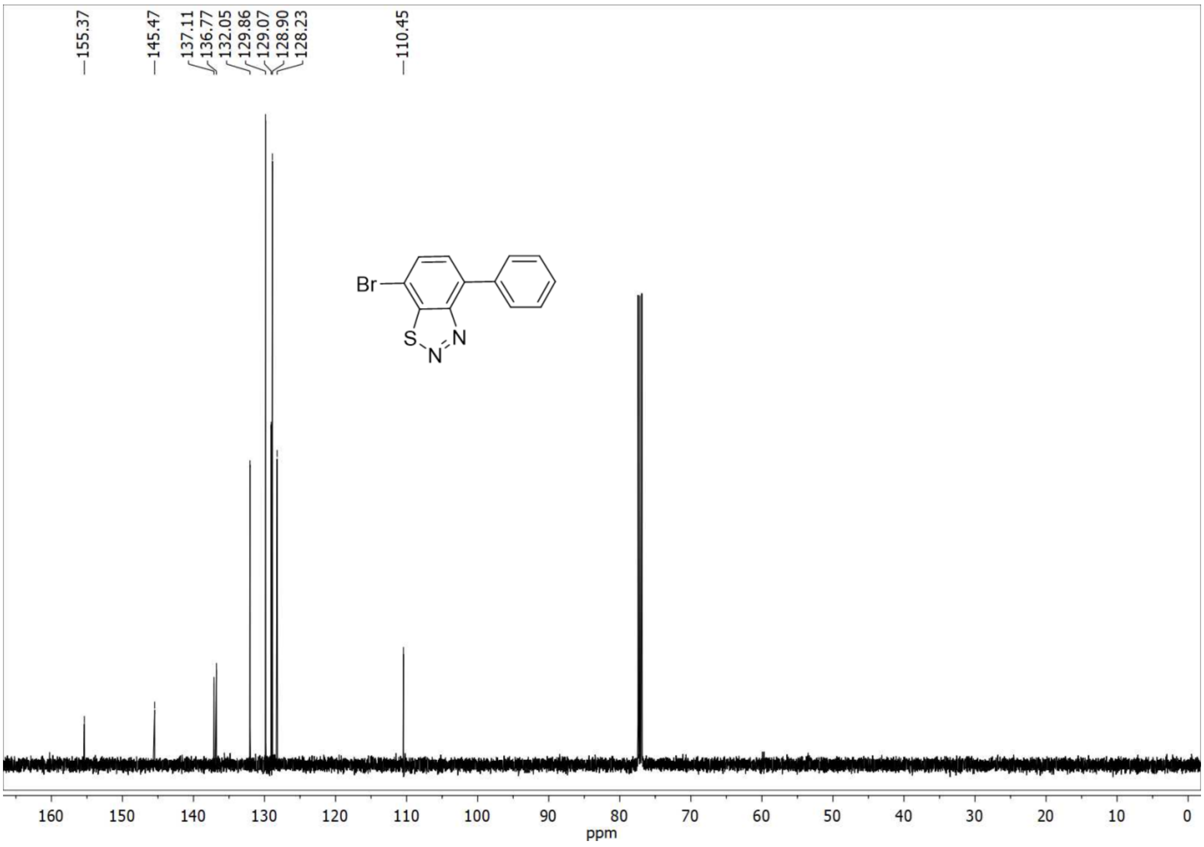

4,7-Diphenylbenzo[*d*][1,2,3]thiadiazole (9e)

^{1}H NMR (300 MHz)

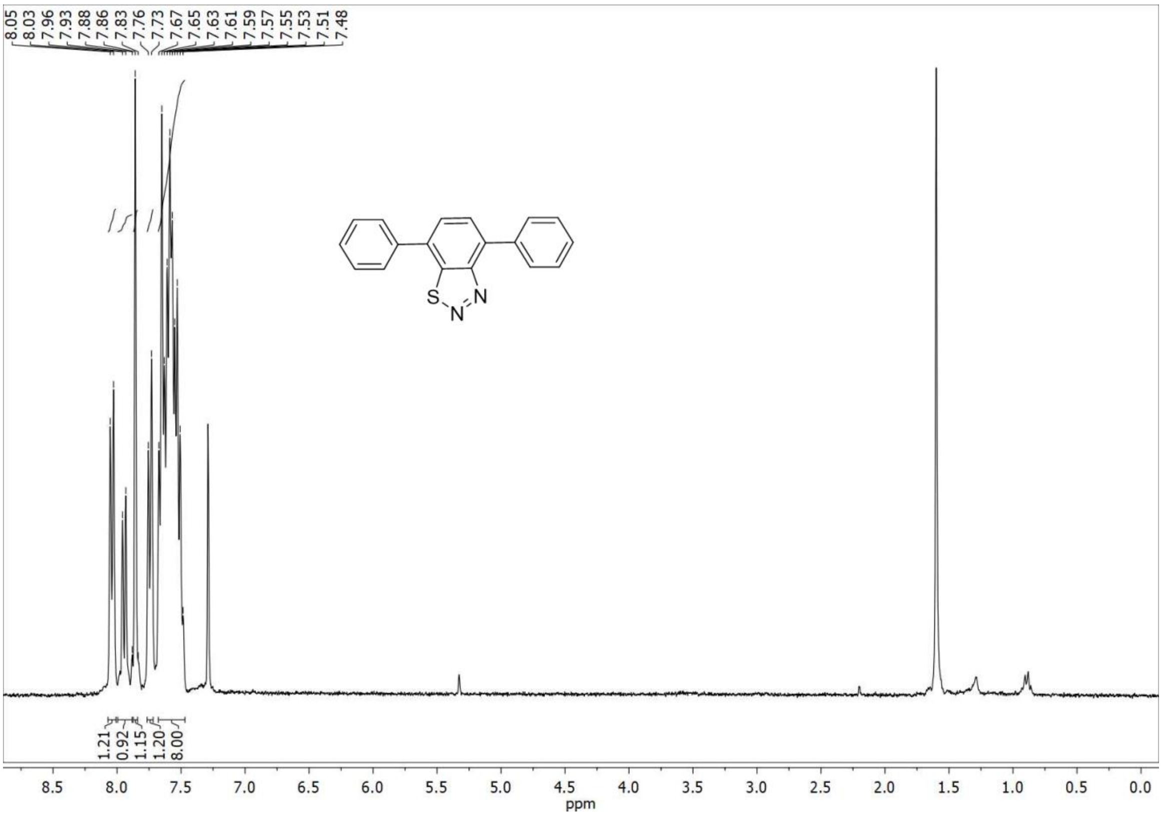

^{13}C NMR (75 MHz)

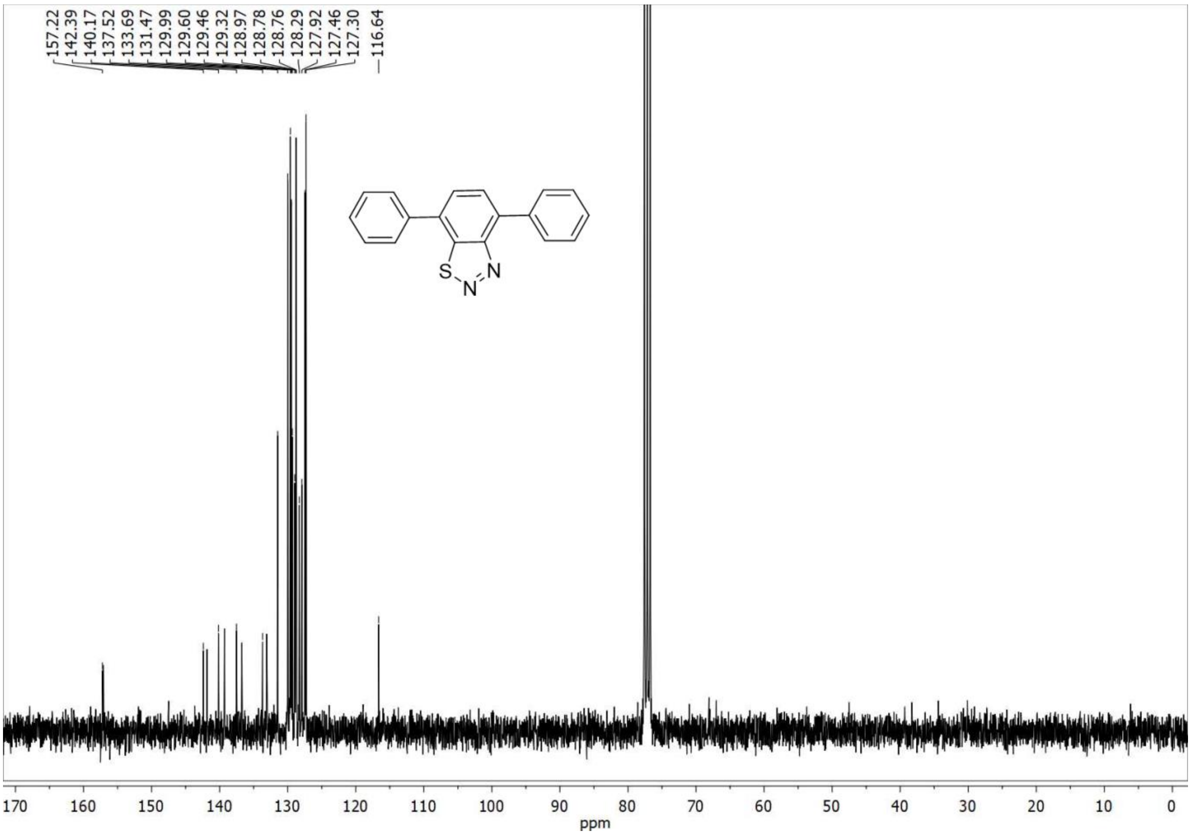

7-Bromo-4-(thiophen-2-yl)benzo[*d*][1,2,3]thiadiazole (7f)

^{1}H NMR (300 MHz)

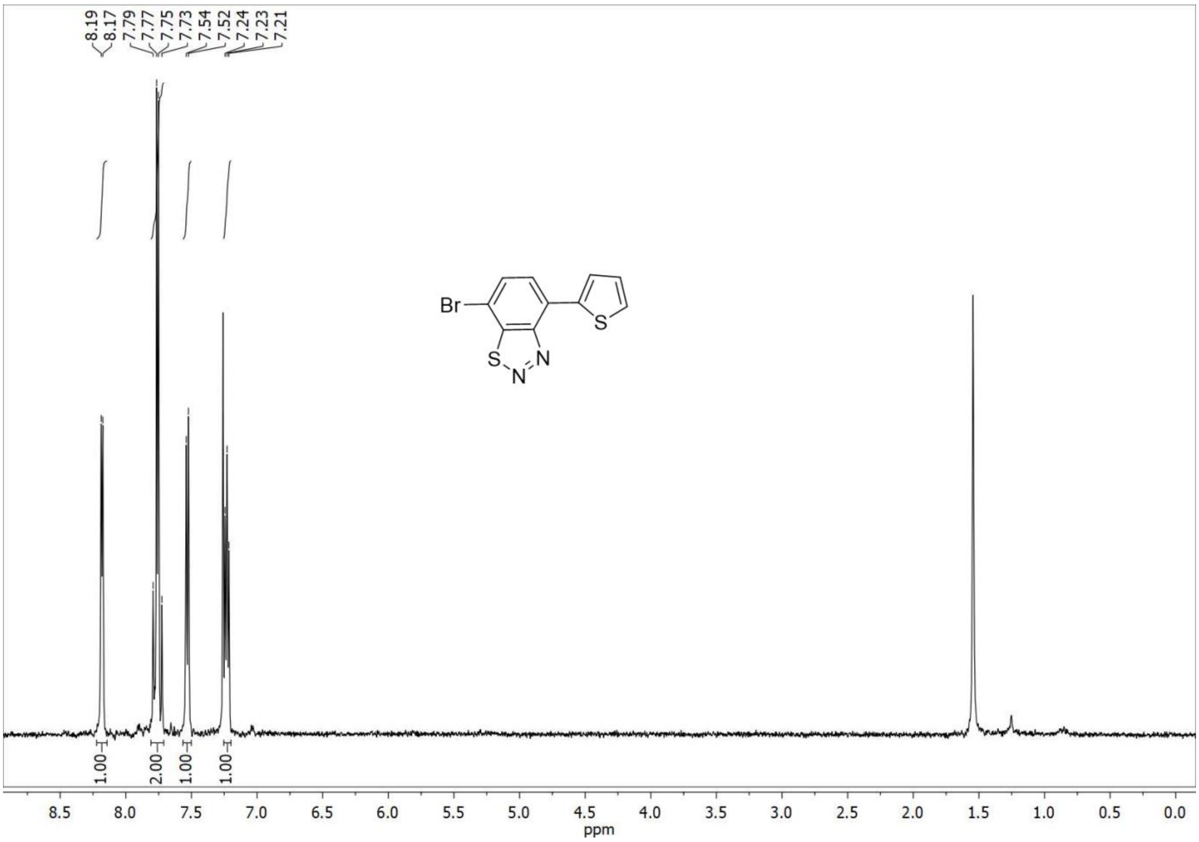

^{13}C NMR (75 MHz)

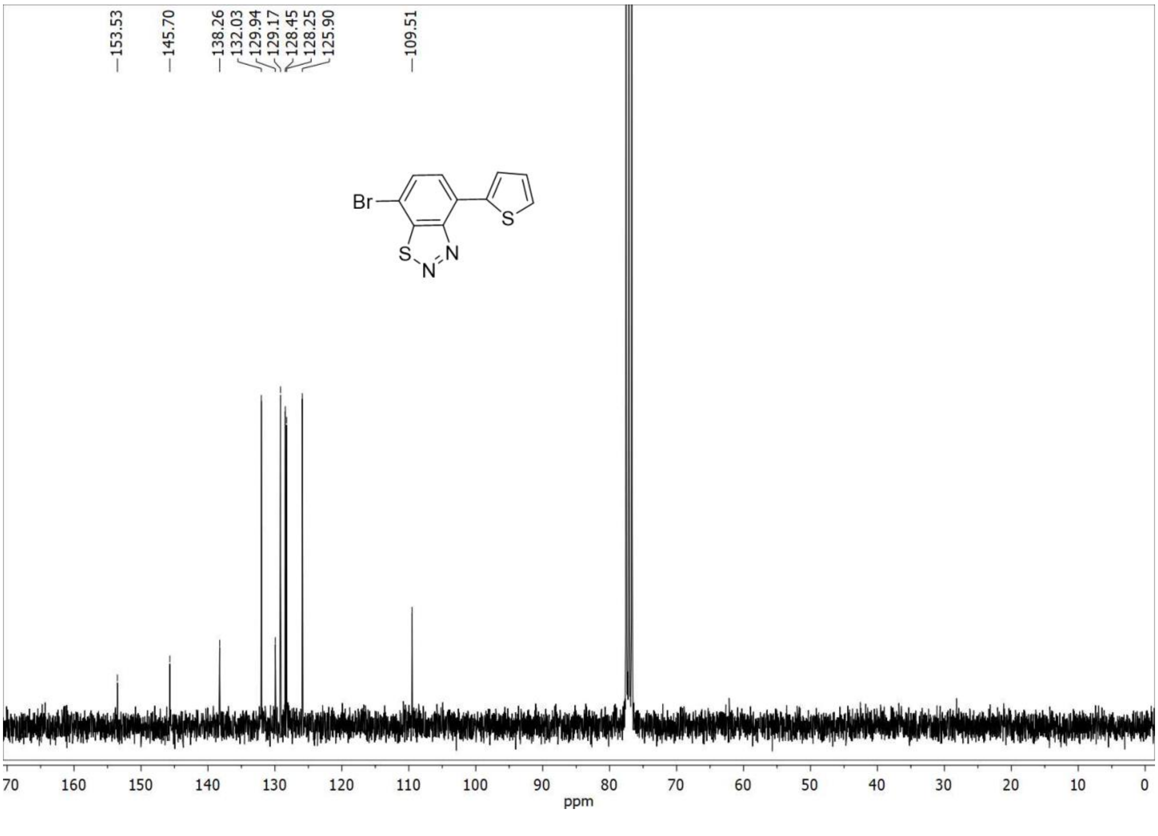

2-Cyano-3-(5-(4-(4-(*p*-tolyl)-1,2,3,3a,4,8b-hexahydrocyclopenta[*b*]indol-7-yl)benzo[*d*][1,2,3]thiadiazol-7-yl)thiophen-2-yl)acrylic acid (KEA321)

^{1}H NMR (300 MHz)

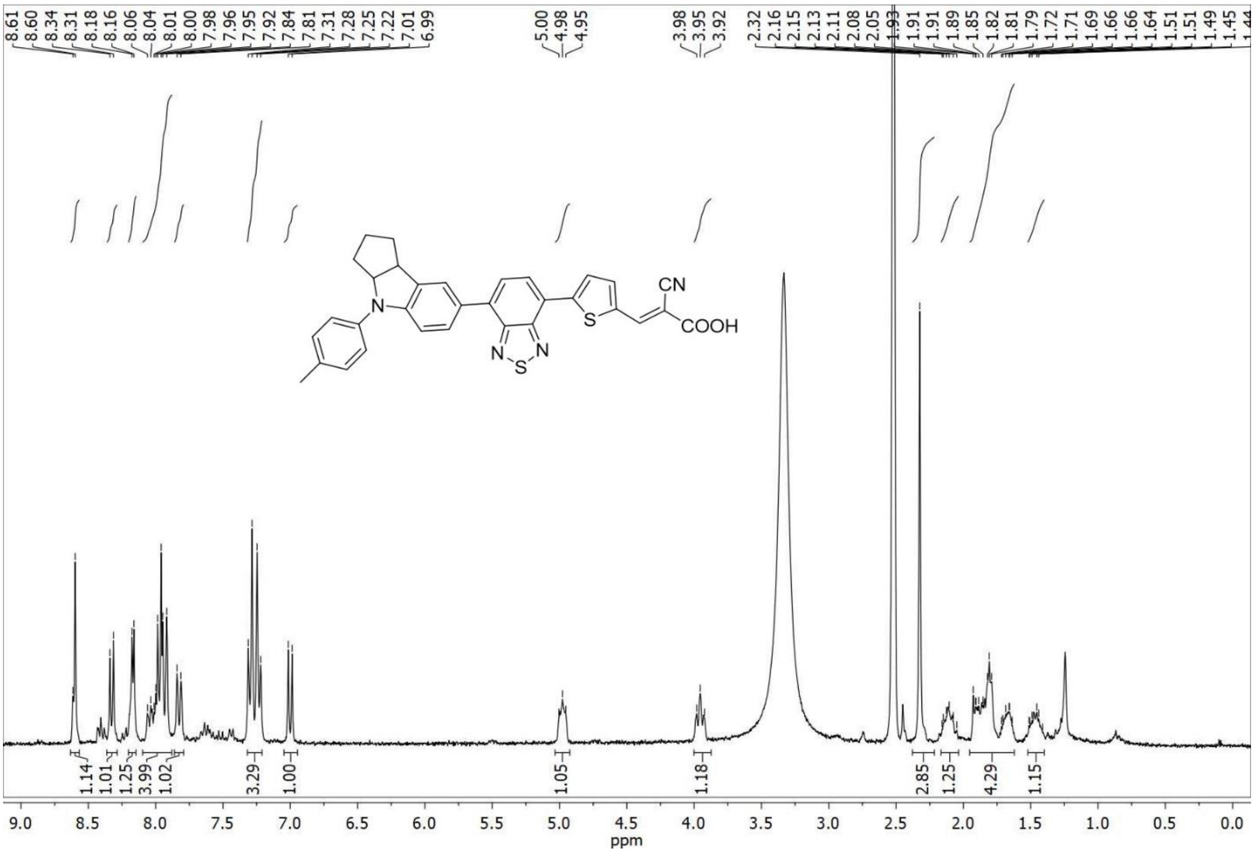

^{13}C NMR (75 MHz)

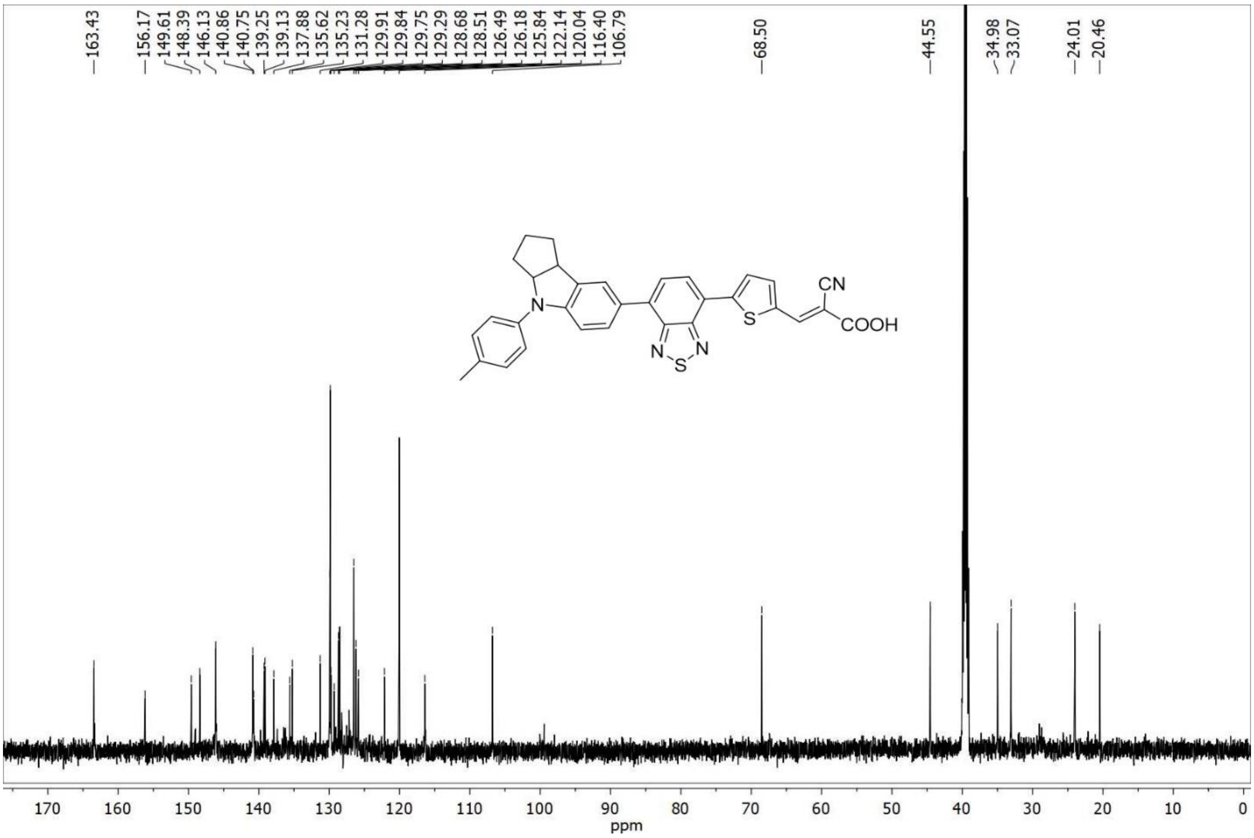

2-Cyano-3-(5-(4-(9-(*p*-tolyl)-2,3,4,4a,9,9a-hexahydro-1*H*-carbazol-6-yl)benzo[*d*][1,2,3]thiadiazol-7-yl)thiophen-2-yl)acrylic acid (KEA337)

^{1}H NMR (300 MHz)

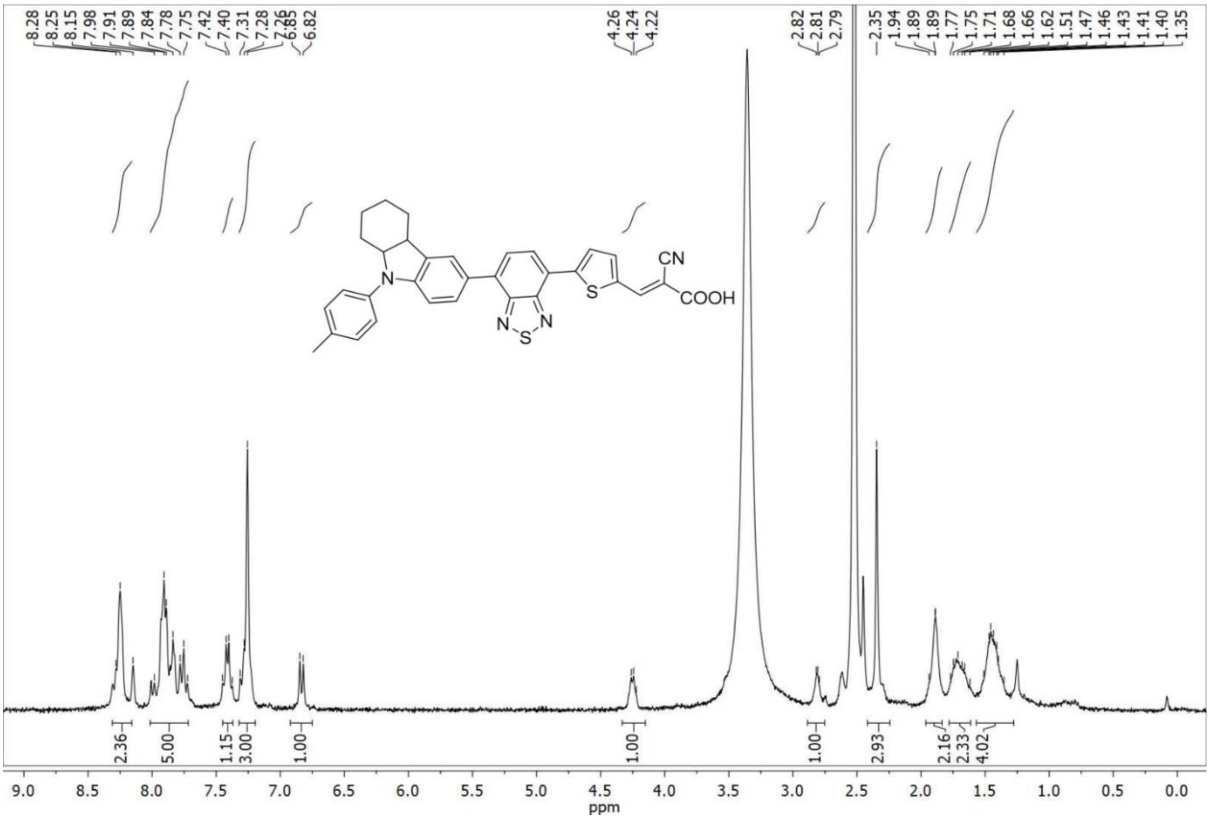

^{13}C NMR (75 MHz)

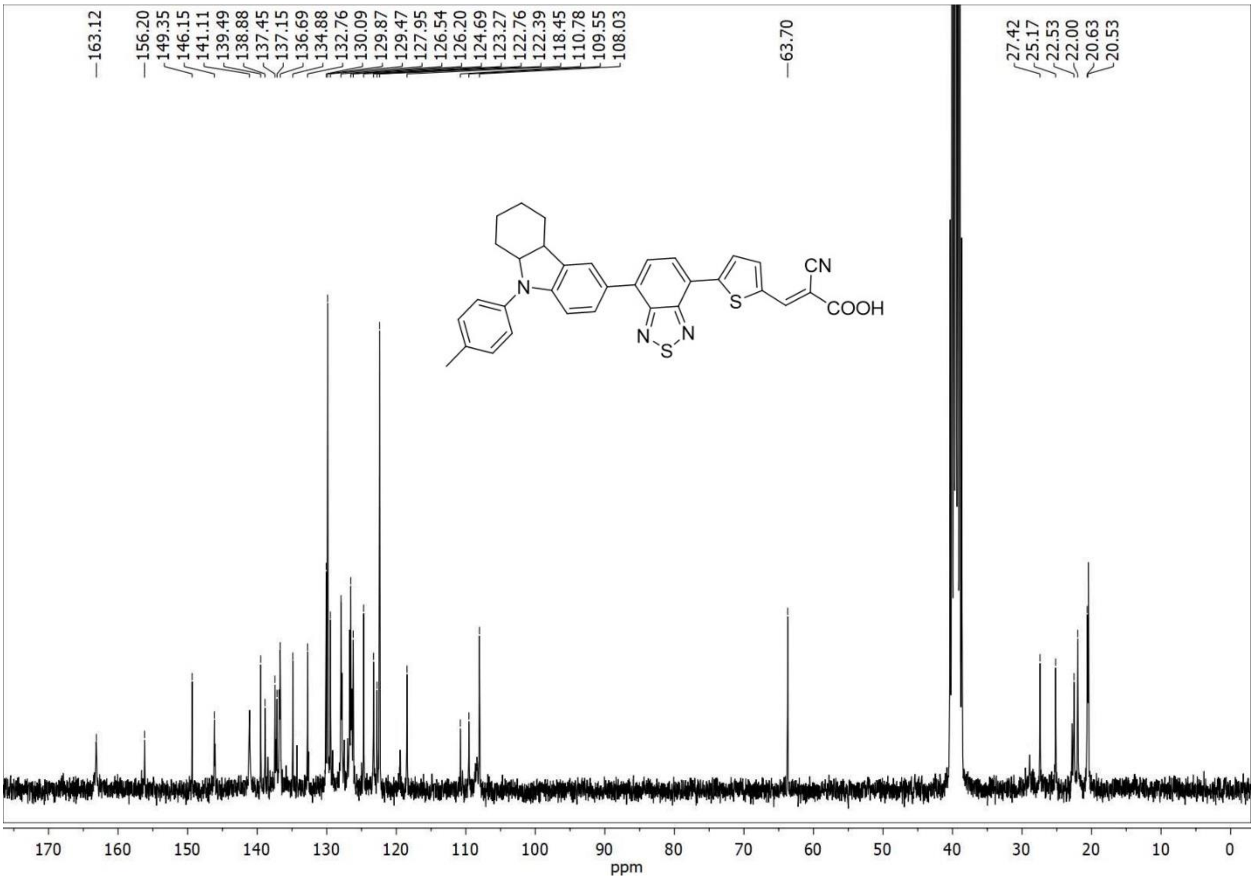

2. Table S1. Crystal data and structure refinement for compound **7e** and **7f**.

	Compound **7e**	Compound **7f**
Empirical formula	$C_{12}H_7BrN_2S$	$C_{10}H_5BrN_2S_2$
Formula weight	291.17	297.19
Temperature	100.00(10) K	100.00(10) K
Wavelength	0.71073 Å	0.71073 Å
Crystal system	monoclinic	monoclinic
Space group	$P12_1/_{c1}$	$P12_1/_{c1}$
Unit cell dimensions	a = 11.4348(3)	a = 3.85910(10)
	b = 14.7803(5)	b = 20.3833(6)
	c = 6.7783(3)	c = 12.7488(5)
	α = 90o	α = 90 o
	β = 106.295(4)o	β = 90.357(3) o
	γ = 90o	γ = 90 o
Volume	1099.58(7) Å3	1002.82(6) Å3
Z	4	4
Density (calculated)	1.759	1.968
Absorption coefficient	3.897	4.476
F(000)	576	584
Crystal size	0.103 x 0.11 x 0.59 mm	0.04 mm3
Theta range for data collection	2.311 to 33.370o	2.559 to 34.479
Index ranges	-16≤h≤16; -22≤k≤19; -10≤l≤9	-6≤h≤5; -31≤k≤32; -19≤l≤19
Reflections collected	3686	3832
Independent reflections	3014 [R(Int) = 0.0522]	3069 [R(Int) = 0.0588]
Completeness to theta	99.94	99.9
Absorption correction	spherical harmonics	spherical harmonics
Max. and min. transmission	(not specified)	(not specified)
Refinement method	Full-matrix least-squares on F2	Full-matrix least-squares on F2
Data / restraints / parameters	3686/0/145	3832/2/146
Goodness-of-fit on F2	1.091	1.029
Final R indices [I>2sigma(I)]	R1 = 0.0367, wR2 = 0.0925	R1 = 0.0389, wR2 = 0.0779
R indices (all data)	R1 = 0.0478, wR2 = 0.0962	R1 = 0.0565, wR2 = 0.0823
Largest diff. peak and hole	1.044 and -0.706 $Å^{-3}$	1.026 and -0.538 $Å^{-3}$

Table S2. Crystal data and structure refinement for compound **7b** and **8b**.

	Compound **7b**	Compound **8b**
Empirical formula	$C_{26}H_{22}BrN_3S$	$C_{26}H_{22}BrN_3S$
Formula weight	488.43	488.43
Temperature	100(2) K	100(2) K
Wavelength	0.71073 Å	0.71073 Å
Crystal system	monoclinic	Triclinic
Space group	$P2_1/c$	P-1
Unit cell dimensions	a = 11.4845(11) Å<br>b = 10.3419(10) Å<br>c = 18.4029(18) Å<br>α = 90°.<br>β = 101.867(3)°.<br>γ = 90°.	a = 9.3264(2) Å<br>b = 9.7012(2) Å<br>c = 12.0096(3) Å<br>α = 78.2162(7)°.<br>β = 89.0002(7)°.<br>γ = 88.9216(8)°.
Volume	2139.0(4) $Å^3$	1063.41(4) $Å^3$
Z	4	2
Density (calculated)	1.517	1.525
Absorption coefficient	2.039	2.051
F(000)	1000	500
Crystal size	0.370 x 0.310 x 0.260 mm	0.570 x 0.300 x 0.120 mm
Theta range for data collection	2.591 to 34.000°	2.467 to 26.998°.
Index ranges	-18≤h≤18, -16≤k≤16, -28≤l≤28	-11≤h≤11, -12≤k≤12, -15≤l≤15
Reflections collected	81377	30186
Independent reflections	8716 [R(Int)= 0.0541]	4636 [R(int) = 0.0311]
Completeness to theta	99.7	99.9
Absorption correction	Multi-scan	Semi-empirical from equivalents
Max. and min. transmission	(not specified)	0.1004 and 0.0595
Refinement method	Full-matrix least-squares on F2	Full-matrix least-squares on F^2
Data / restraints / parameters	8716/0/281	4636 / 1 / 281
Goodness-of-fit on F2	1.014	1.073
Final R indices [I>2sigma(I)]	R1 = 0.0333, wR2 = 0.0909	R1 = 0.0719, wR2 = 0.1870
R indices (all data)	R1 = 0.0418, wR2 = 0.0980	R1 = 0.0781, wR2 = 0.1928
Largest diff. peak and hole	0.823 and -0.622 $Å^{-3}$	2.446 and -1.031 e.$Å^{-3}$

3. Statistics of photovoltaic performance of DSSCs fabricated with KEA dyes

Dye	V_{oc} (mV)	J_{sc} (mA·cm^{-2})	FF	η (%)
**KEA321**	0.65	12.94	0.61	5.15
	0.74	5.09	0.68	2.54
**KEA337**	0.66	10.99	0.67	4.83
	0.68	10.32	0.67	4.69

4. General procedure for fabrication and characterization of DSSCs

Clean fluorine doped tin oxide (FTO) conductive glass substrates (7 Ω/□, 2.2 mm-thick, TCO22-7/LI, Solaronix) were treated with 40 mM $TiCl_4$ aqueous solution at 80 °C for 30 min on a hotplate. Transparent and scattering TiO_2 pastes (18NR-T and 18NR-AO, then Greatcell Solar, now Greatcell Solar Materials) were deposited on the treated substrates, one layer each, by screen printing. The TiO_2 electrodes were annealed in a furnace at 325 °C for 5 °C min, 375 °C for 5 min, 450 °C for 15 min and 500 °C for 15 min (ramp: 10 °C/min), resulting in ~8 μm-thick (4 μm + 4 μm) mesoporous TiO_2 films. The films were treated for a second time in $TiCl_4$ solution, followed by annealing at 500 °C for 30 min. The electrodes were immersed in the dye bath while still warm (~80 °C). The dye baths consisted of 0.3 mM concentration in toluene/EtOH (v/v, 7/3). The dye adsorption was performed overnight. For the counter electrodes, a platinum precursor paste (Platisol T, Solaronix) was doctor-bladed on clean FTO glass substrates and fired at 450 °C for 10 minutes. The anode and cathode were assembled with a 25 μm-thick thermoplastic gasket (Surlyn, DuPont), and the electrolyte was injected under vacuum through a predrilled hole in the cathode. The electrolyte in our study consisted of 0.6 M 1,2-dimethyl-3-propylimidazolium iodide, 0.1 M LiI, 0.1 M iodine and 0.5 M 4-*tert*-butylpyridine in anhydrous acetonitrile.

Current-voltage characteristics were recorded on a potentiostat (Autolab, Metrohm) equipped with AM1.5G (100 mW cm^{-2}) simulated sunlight as light source, generated by a class AAA solar simulator (SLB300A, Sciencetech). The intensity of the incident light was calibrated with a Si reference cell. The active area of the solar cell was fixed to 0.0625 cm^2 by a black metal mask.
